# Gecko-Inspired Intelligent Adhesive Structures for Rough Surfaces

**DOI:** 10.34133/research.0630

**Published:** 2025-02-25

**Authors:** Yawen Shao, Miao Li, Hongmiao Tian, Fabo Zhao, Jian Xu, Hongrong Hou, Zhijun Zhang, Duorui Wang, Xiaoliang Chen, Wenjun Li, Hongjian Yan, Jinyou Shao

**Affiliations:** ^1^ State Key Laboratory for Manufacturing Systems Engineering, Xi’an Jiaotong University, Xi’an, China.; ^2^ Caihong Display Devices Company Limited, Xianyang, China.; ^3^ Frontier Institute of Science and Technology (FIST), Xi’an Jiaotong University, Xi’an, China.

## Abstract

Biomimetic dry adhesive structures, inspired by geckos’ climbing abilities, have attracted research attention in recent years. However, achieving superior adhesion on a rough surface remains an important challenge, which limits practical applications. Conventional bionic adhesion methods perform well on smooth surfaces, but adhesion strength drastically decreases on rough surfaces due to the reduced contact area. Generally, various adhesive structures have been proposed to increase the contact area without assessing adhesion states, against obtaining good performance on rough surfaces. If an intelligent adhesive approach could be introduced on rough surfaces, it would be beneficial for promoting the development of gecko-inspired adhesives. However, existing adhesive structures with the sensing function usually utilize the adhesive function to support the sensing function, i.e., a sensor with an adhesive function; for other few structures, the sensing function supports adhesion, but they do not focus on improving adhesion performance on rough surfaces. Inspired by the synergistic effect of a kinematic system during the crawling process of geckos, this study proposes an intelligent adhesive structure for rough surfaces. The proposed structure combines a hierarchical bionic dry adhesive structure based on gecko paw microhairs with a flexible capacitive sensor unit. Experimental observations and analytical modeling demonstrate that incorporating mushroom-shaped bionic dry adhesive structures with inclined support micropillars can reduce interface contact stiffness, notably enhancing adhesion on rough surfaces while allowing real-time monitoring of contact states. Moreover, this innovative smart adhesive structure facilitates morphology sensing of contact interfaces, presenting potential advancements in bionic adhesion for morphology sensing applications.

## Introduction

In nature, small organisms, such as beetles, spiders, and geckos, can freely crawl in complex environments due to their unique adhesion properties of their feet [1–4]. Geckos, as a representative of such organisms, have adhesion properties far exceeding those of traditional pressure-sensitive adhesives [5,6], which has inspired the development of bionic dry adhesive structures [5–9]. These structures have been widely applied to various systems, including production lines, robotic arms, and health monitoring systems [10–12]. They have also significantly improved the ability of conveying, grasping, attaching, and climbing and maintained efficient adhesion in extreme environments, such as space [13–16]. Due to all these benefits, researchers have been continuously exploring the bionic structure at the end of the gecko’s paw, which provides notable advantages over the application of traditional adhesion techniques [17]. First, it ensures clean and residue-free physical adhesion, avoiding the residues that might be left behind by chemical liquid adhesives. Second, its adhesion and detachment processes are extremely rapid. Third, the bionic dry adhesive structure is easy to integrate, does not rely on external field sources, and can be flexibly combined with a variety of active devices. Fourth, it has a wide application range in various environments without requiring specific electromagnetic and other external field assistance. Finally, it performs adhesion tasks consistently and with good reproducibility in a wide range of common environments.

However, the practical application of bionic adhesive structures in engineering environments faces many challenges. First, most of the studies of bionic adhesive structures have mainly considered smooth surfaces, whereas adhesion environments in real engineering application scenarios are typically more complex [18–20]. Also, bionic adhesive structures perform relatively well on smooth surfaces, but their effective contact area decreases significantly with the increase in surface roughness [21–23], which can cause a noticeable decrease in adhesion. Second, monitoring and controlling the pre-pressure while maintaining it at an appropriate value have an important influence on a bionic adhesive structure’s performance. In most bionic adhesive materials, the adhesion forces, both normal and tangential forces, increase with the pre-pressure. If the pre-pressure is too low, the surface cannot be adhered to, whereas an excessive pre-pressure might disrupt the microstructure of a material and limit the further enhancement of the adhesion force. Further, it is difficult to observe the shedding state directly during the adhesion process. Bionic adhesive structures often involve a micrometer or nanoscale fabrication process, so their integrity and functionality need to be maintained, making nondestructive testing necessary. Moreover, the requirement for a tight fit to the contact surface during the adhesion process makes the localized shedding process difficult to monitor visually. Furthermore, changes in environmental conditions can affect the size and stability of the adhesion force, thus increasing the difficulty of predicting and monitoring shedding during the adhesion process. Therefore, to promote the development of bionic bonding technology and its application in a wider range of fields, in-depth research on the adhesion technology of rough surfaces and the dynamic monitoring technology during the adhesion process is required [24].

To optimize the adhesion to rough surfaces, many hierarchical bionic adhesive structures have been proposed in recent years [25–27]. These structures do not depend on the external field action, and by changing the shape of the structure, the modulus of elasticity and the adaptability of the whole system, which is necessary to enhance the adhesion of rough surfaces, can be improved [28–33]. However, most recent studies have not been able to achieve real-time monitoring of the adhesion state while improving environmental adaptability. Nonetheless, with the development of flexible sensors, the integration of sensing technology and adhesive structures has attracted widespread research attention. Compared to traditional dry adhesion, intelligent and controllable adhesion is more adaptable to complex and diverse target surfaces, which can better meet the requirements of industrial, medical [34–36], and intelligent wearable fields [37,38]. Therefore, many studies have introduced sensing functions into adhesive structures to realize real-time monitoring and control of the adhesion performance. Still, most research has focused on the independent design of sensing and adhesion functions, and the adhesion function often exists only as an auxiliary means of sensing [30,39], which limits substantial improvement in adhesion performance and controllability. Nevertheless, certain research attempts have been made to combine adhesion and sensing to design integrated structures [40], but these structures have not made marked breakthroughs in enhancing adhesion performance yet, and their adhesion force to rough surfaces needs further improvement. Motivated by previous studies, this study focuses on the in-depth integration of sensing and adhesive structures and proposes an innovative type of intelligent adhesive structure that can sense the adhesion state in real time intelligently and adapt to a wide range of surfaces.

**Fig. 1.**
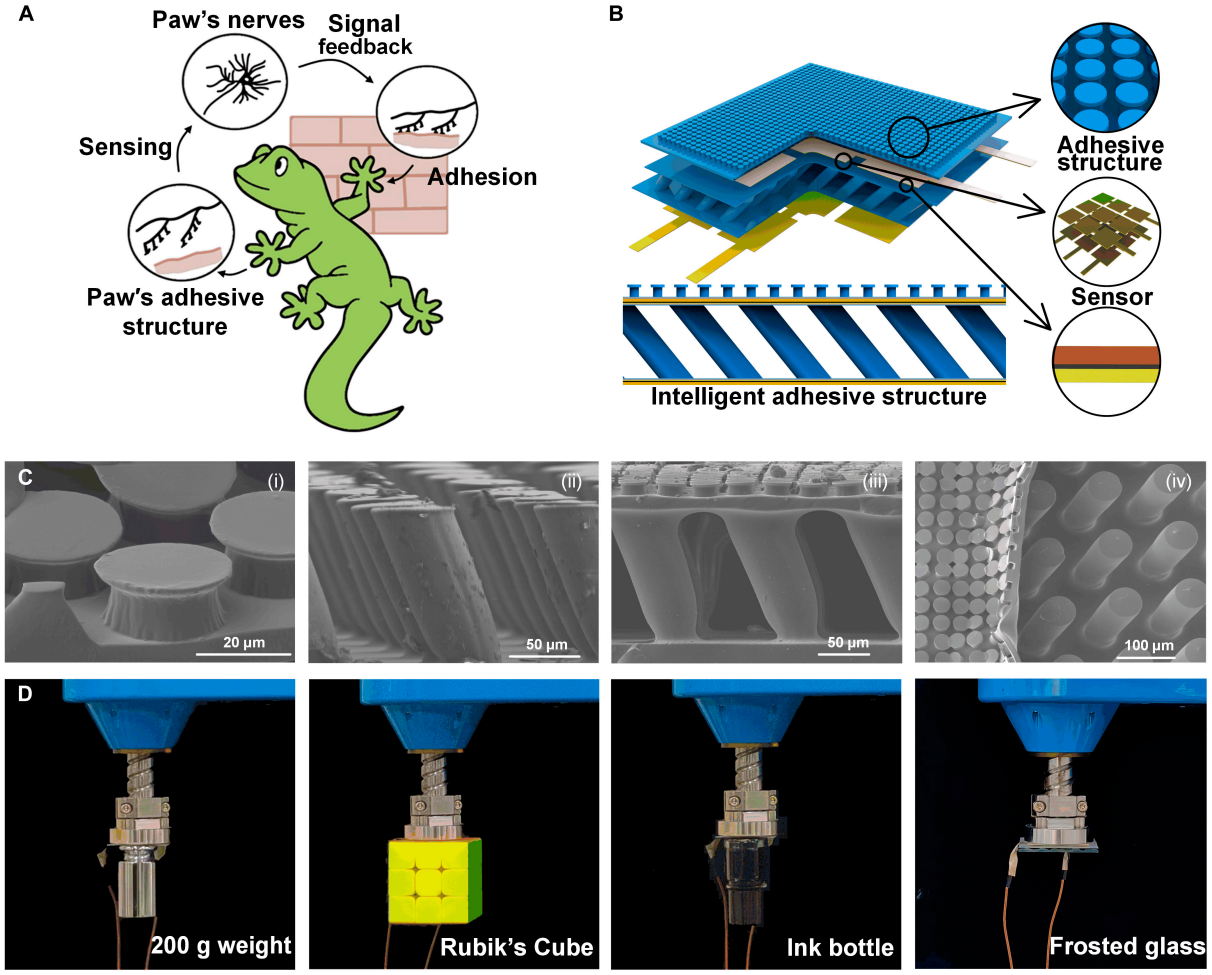
Gecko-inspired hierarchical structures in the intelligent adhesive structure. (A) The crawling process of a gecko realized by joint action of the adhesion system of its toes and nervous system. (B) An integrated adhesion/sensing structure consisting of a mushroom structure and a tilted micropillar support structure with hierarchical bionic dry adhesion properties, which support the adhesive electrodes on both sides to sense capacitance changes for sensing functions. (C) The scanning electron microscopy (SEM) image of the integrated adhesion or sensing structure: (i) a mushroom-type adhesive structure; (ii) a bottom tilted micropillar support structure; (iii and iv) the side and top views of a hierarchical bionic dry adhesive structure. (D) Diagrams of pickup experiments of hierarchical bionic dry adhesive structures with smooth surfaces (200-g weight and Rubik’s Cube), concave surfaces (the bottom of an ink bottle), and rough surfaces (frosted glass).

Fortunately, a gecko’s paws have a unique function, as shown in Fig. 1A, where the adhesion process is accompanied by neural feedback and regulation during the gecko’s climbing process, which is crucial for achieving an intelligent adhesion effect. A gecko’s nervous system is responsible for the sensing function and receiving information on the surrounding environment, such as the roughness and inclination of a surface, as well as for processing and analyzing this information and sending precise commands to the adhesion system of the paws accordingly [8,41–43]. This immediate feedback mechanism allows a gecko to adjust the adhesion state of its paws flexibly to adapt to a changing climbing environment. Inspired by the synergy between the adhesion system and the neural system of geckos during the climbing process, this study proposes an intelligent adhesive structure for rough surfaces, which is presented in Fig. 1B. The proposed structure can not only enhance the adhesion ability on rough surfaces by adopting a hierarchical bionic design [44–47] but also integrate flexible capacitive sensors to realize real-time monitoring and regulation of the interface contact state. In concrete terms, this study introduces a mushroom-type biomimetic dry adhesive structure with excellent adhesion ability to imitate a gecko’s claw end in the top layer [47–51]. Further, the problem of decreased adhesion on rough surfaces caused by the limitation of the effective contact area is solved by adding an inclined support structure to the bottom layer. This elastic support structure deforms when it encounters a rough surface [23], reducing the stiffness of the contact interface and increasing the area of the adhesive structure in contact with the target surface. In addition, the inclined support structure can be used as part of a capacitive sensor for detecting the interfacial contact state of the integrated smart adhesive structure. The results show that the hierarchical bionic dry adhesive structure has better adaptability on rough surfaces compared to the normal adhesive structure. Finally, this study also prepares a regionalized intelligent adhesive structure using flexible capacitive sensors, and each region of the regionalized intelligent adhesion structure detects the corresponding capacitance change and then feeds back the force. In addition to the interface morphology that can be sensed during the bonding process shown in the paper, subsequent processing procedures can be designed according to practical needs, such as determining whether the pre-pressure is appropriate or not and making changes based on the real-time data on the amount of bonding force obtained by the intelligent adhesion structure.

## Results and Discussion

Geckos use the hierarchical structure of their feet to make contact with target surfaces, resulting in strong adhesion. Each small hair consists of multiple branches and small end structures that increase the actual area of contact with the surface, enhancing adhesion [52]. Inspired by a gecko’s paw, this study proposes a design of adding a flexible substrate on the back of a bionic dry adhesive structure to mimic the flexibility of gecko bristles, aiming to improve the adhesion ability on rough surfaces. Compared to the traditional hierarchical structures that face the problems of low mechanical strength and a small effective adhesion area, the proposed hierarchical bionic dry adhesive structure combines an inclined support structure with a mushroom-type top adhesive structure, which is flexed by the connection of flexible films to adapt to a rough surface and increase the contact area to improve the adhesion effect. A scanning electron microscopy image of the proposed structure is presented in Fig. 1C, where sub-image (i) displays the top mushroom-type biomimetic dry adhesive structure that realizes high-density contact and strong adhesion performance with a contact surface. Cylindrical arrays with mushroom-type tip morphology are used to achieve high-density contact with a contact surface and achieve the accumulation of van der Waals forces. The synergistic effect of these microstructures and nanostructures at different scales enhances the stability and persistence of adhesion. Sub-image (ii) shows a scanning electron microscopy image of the underlying support structure. The inclined support structure of the underlying layer provides reliable support for the adhesion layer, which can facilitate the efficient execution of the adhesion operation. Sub-figures (iii) and (iv) display the side and top views of a hierarchical bionic dry adhesive structure, respectively, where it can be seen that the top layer of the adhesive structure and the bottom layer of the support structure form a tight and seamless monolithic connection after being precisely bonded together; this connecting mechanism ensures extreme robustness of the structure. After the 2 layers are bonded into a single unit, the original layer of a flexible film between the 2 structures can be bent in response to the interfacial contact, which is functionally similar to a gecko’s bristles, thus enhancing the adhesion effect on rough surfaces. Figure 1D demonstrates the adhesion effect of the hierarchical bionic dry adhesive structure when facing objects with different surface characteristics. The results show that the proposed hierarchical bionic adhesive structure has excellent adaptability to objects with smooth surfaces (e.g., weights and Rubik’s Cube). In addition, sufficient adhesion ability was also demonstrated when grasping objects with concave bottoms (e.g., ink bottles) and on rough surfaces (e.g., frosted glass). The frosted glass sample and its confocal features are described in Fig. S1.

The use of pillar structures as the supporting layer in a hierarchical structure offers advantages in the following aspects. Firstly, unlike foam and gel materials, the pillar structure features a well-defined geometry, and its inherent relationship between pressure and deformation can be precisely calculated using mechanical models. This allows for the establishment of a correlation between sensor performance and structural configuration, such as the relationship between capacitance change and the degree of bending under pressure. This structure–property relationship is closely related to factors such as material properties, pillar bending angle, and pillar height, enabling customized design solutions for various application scenarios and facilitating reverse engineering to meet specific requirements. These characteristics are not found in foam and gel materials. Furthermore, because the material used in the various parts of the hierarchical bionic dry adhesive structure is mostly the same when the pillar structure is used, it can be regarded as a seamless and complete structure with greater mechanical strength and stability without the need for seam treatment and without potential quality problems. The pillar structure also outperforms foam and gel materials in terms of durability and antiaging properties, maintaining stable mechanical performance and adhesion over extended periods of use, whereas foam and gel materials are prone to aging, hardening, or losing elasticity, which can compromise their reliability. Additionally, pillar materials demonstrate greater adaptability to environmental changes, such as temperature and humidity fluctuations, with performance that is less affected by environmental variations. In contrast, gel materials are more sensitive, particularly when stability is required over long periods or under harsh environmental conditions.

**Fig. 2.**
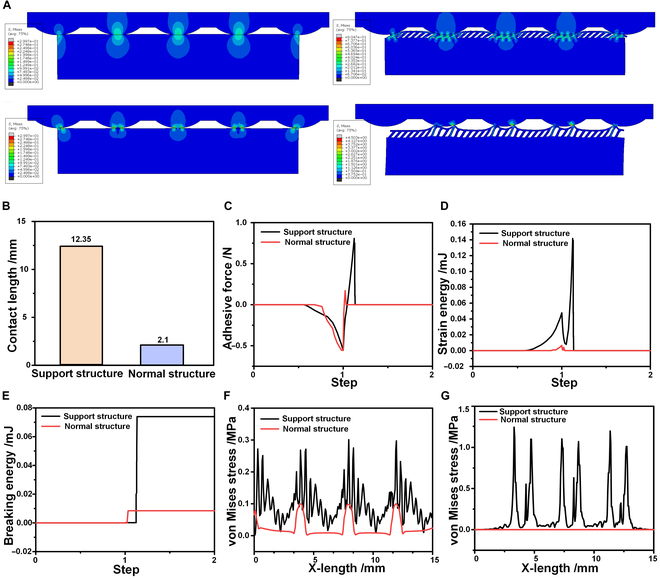
Simulation and verification of interface contact laws for hierarchical structures. (A) The simulation model of the adhesion effect on a rough surface; the left side shows the simulation of the adhesion process of the ordinary dry adhesive structure with a rough surface, and the right side displays the simulation of the adhesion process of the proposed hierarchical bionic dry adhesive structure with a rough surface. The upper figure shows the state at the maximum pre-pressure at the down-pressure stage, and the lower figure displays the state at the maximum adhesion force at the separation stage. (B) The comparison graph of the maximum effective contact area during the simulation process. (C) The comparison graph of the adhesion force during the simulation process. (D) The comparison graph of the strain energy during the simulation process. (E) The comparison graph of the breaking energy during the simulation process. (F) The comparison plot of the von Mises stress at the maximum preload. (G) The comparison plot of the von Mises stress at the maximum adhesion force.

To simulate and verify the interfacial contact law of hierarchical structures, this paper constructed a relevant physical model of adhesion based on a cohesion model and an interfacial fracture damage criterion and verified through simulation experiments that the hierarchical structures could better adapt to the target surfaces than the ordinary structures due to the application advantages of adhesion [53–55]. The difference in the adhesion ability between the proposed hierarchical structure and the normal structure was analyzed, and more details about the exact modeling process can be found in Figs. S2 and S3. Figure 2A shows 2 experimental groups used in the simulation process, namely, the rough surface adhered with the normal bionic dry adhesive structure and the rough surface adhered with the hierarchical bionic dry adhesive structure. In the experimental model, the upper part was set as a rough surface, and in the lower part was placed the normal bionic dry adhesive structure and hierarchical bionic dry adhesive structure. The experimental process is divided into 2 stages. The first stage was the gradual approach of the adhesive structure to the test surface until it reached the set position, during which a gradually increasing pre-pressure was generated. The second stage involved the adhesive structure gradually moving away from the target surface until the adhesive structure separated from the test surface. (The complete simulation process is described in Movies S1 and S2.)

In the process of pressure–tension transition, the hierarchical bionic dry adhesive structure showed a clear advantage in terms of adhesion performance on rough surfaces, and its contact area was significantly larger than that of the ordinary bionic dry adhesive structure. As the previous theoretical analyses showed, the reason for such a sharp decrease in the adhesion force of the normal bionic dry adhesive structure on the rough surface was the decrease in the effective contact area. As presented in Fig. 2B, the effective contact area of the hierarchical bionic dry adhesive structure facing the rough surface was as high as 12.35 mm, whereas that of the ordinary bionic dry adhesive structure on the rough surface was only 2.1 mm, which indicated a reduction of 82.996%. When the hierarchical bionic dry adhesive structure faced the rough surface, its effective contact area was significantly larger than that of the conventional bionic dry adhesive structure, meaning that the hierarchical bionic dry adhesive structure was more advantageous in design and could better adapt to and fit the rough surface; this provided stronger adhesion and a more stable gripping effect. Figure 2C shows the comparison results of the adhesion simulation under a constant pre-pressure condition. The results showed that the maximum adhesion force of the conventional bionic dry adhesive structure was significantly lower at 0.17 N, whereas the maximum adhesion force of the hierarchical bionic dry adhesive structure was as high as 0.81 N, which indicated that it had a more impressive adhesion performance under the same conditions.

Strain energy and fracture energy are usually used as key indicators when analyzing adhesion properties from the perspective of energy analysis. Strain energy is the energy stored in a material due to deformation, and an increase in this energy implies a decrease in the modulus of elasticity of a structure and an important deformation, which can inhibit crack propagation and enhance adhesion through morphological changes. By contrast, fracture energy is the energy consumed in the process of interfacial separation from the intact to complete fracture, and its value directly indicates the intensity of the adhesion force; namely, the larger the fracture energy’s value is, the more notable the adhesion force and adhesion effect will be. The simulation results verify that the hierarchical bionic dry adhesive structure significantly outperformed the conventional structure in terms of both strain energy (Fig. 2D) and fracture energy (Fig. 2E); the hierarchical bionic dry adhesive structure had a superior adhesion performance when facing rough surfaces.

Two time points were selected for comparison of the von Mises stress. One point denoted the last point of the active contact stage, which indicated that the maximum pre-pressure was reached at this point; also, the stress at this point was the maximum stress at the pre-pressure application stage. Another point was the first calculation point when the maximum adhesion was reached at the detachment stage; the stress at this point was the maximum stress at the detachment stage. As shown in Fig. 2F, when the hierarchical bionic dry adhesive structure was in contact with a rough surface (i.e., the peaked structure), there was a notable increase in the von Mises stress caused by additional deformation; however, the conventional structure exhibited a more uniform stress distribution. As displayed in Fig. 2G, at the pull-up phase, the characteristics of each wave peak were highly consistent with the target surface morphology, which indicated that the adhesion force was mainly concentrated in the contact area of the wave peaks; however, the stress change in the uncontacted part was not marked. This phenomenon was in line with the previous discussion on the contact area and emphasized that under the conditions of a complex rough surface, the hierarchical bionic dry adhesive structure could increase the effective contact area by virtue of its excellent adaptability; this could significantly enhance the adhesion force and adhesion effect. In contrast, the conventional structure had difficulty handling surface properties effectively.

**Fig. 3.**
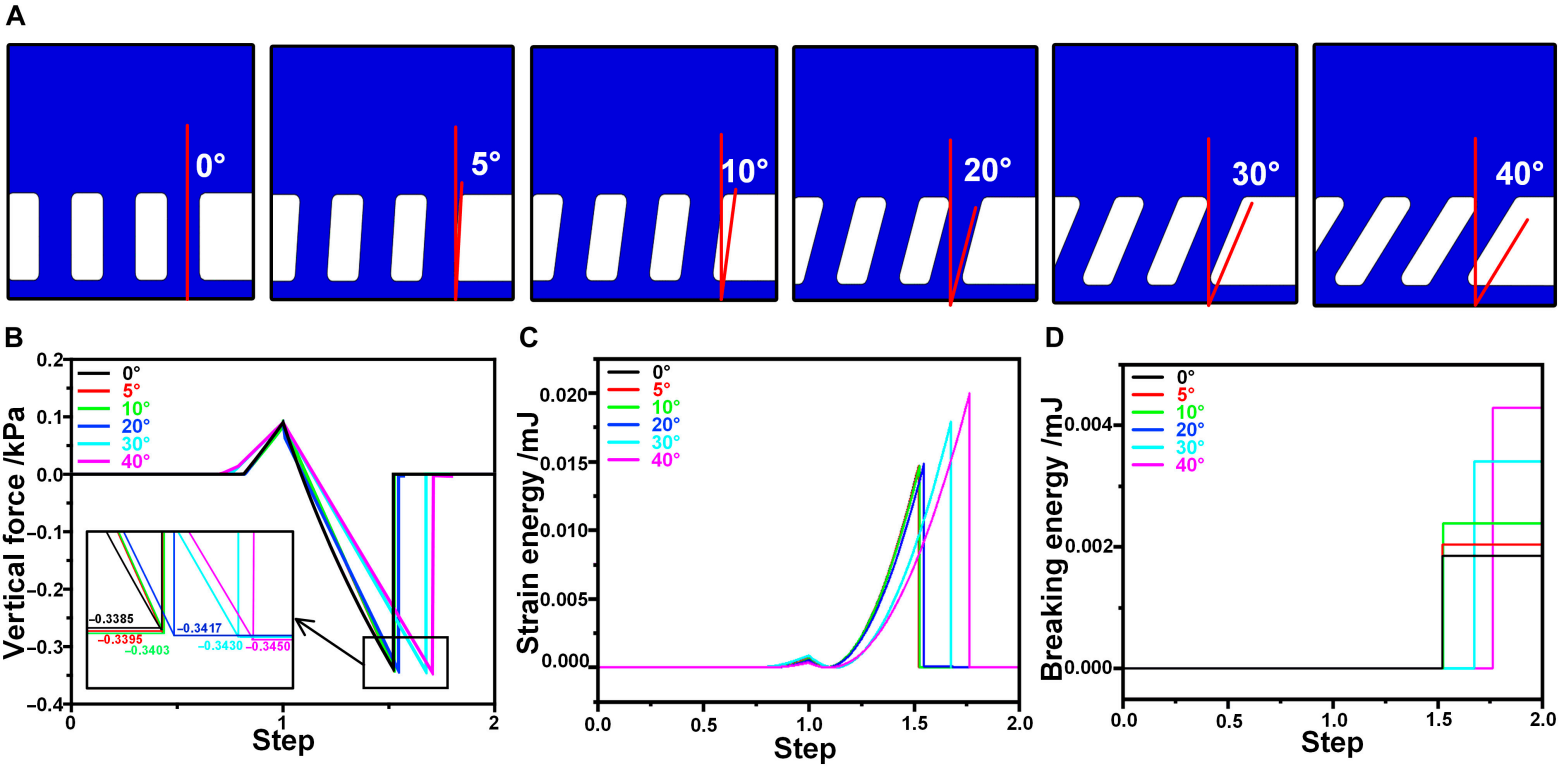
The simulation for optimizing the support layer of the hierarchical structure. (A) Schematic of the modeling of the hierarchical bionic adhesive structure with different angles of the support layer. (B) Adhesive forces generated by a hierarchical adhesion structure with inclined support layers at various angles under the same pre-pressure. (C) The comparison of strain energies of hierarchical adhesive structures with different angles of the support layer during simulation. (D) The comparison graph of the breaking energy of hierarchical adhesive structures with different angles of the support layer during simulation.

After confirming that the hierarchical bionic adhesive structures exhibited superior adhesive performance compared to normal adhesive structures, this study further designed a simulation experiment to investigate how the adhesive performance changed under supporting structures tilted at different angles. The aim was to determine the optimal tilt angle for the support layer in hierarchical adhesive structures. Under the condition that other factors remained constant, the simulation model shown in Fig. 2A was used as the basis for adjusting the tilt angle of the support layer, setting the angles at 0°, 5°, 10°, 20°, 30°, and 40°, as depicted in Fig. 3A. The tilt angle referred to the angle between the supporting structure and the vertical surface. Larger tilt angles were not considered due to observations in the experiments that, when the tilt angle of the support structure was too large, it lacked stability under external forces, causing the pillars to collapse and negatively affecting the overall performance of the structure.

As shown in Fig. 3B, under the same pre-pressure, the bonding force showed an increasing trend with the increase in the tilt angle of the support structure. An energy-based analysis was then conducted, and Fig. 3C and D illustrate the changes in strain energy and breaking energy, respectively. Under the same contact conditions, the support structures with different tilt angles exhibited different strain energy characteristics, with higher strain energy and breaking energy values corresponding to larger tilt angles. When the tilt angle of the supporting structure reached 40°, both strain energy and fracture energy reached their maximum values, and the adhesive performance was also optimal. This phenomenon could be attributed to 2 main factors: first, the tilted support structure, when subjected to external loads, behaved similarly to springs. As it deformed, it rebounded the applied pressure back to the adhesive structure according to Hooke’s law. The adhesive structure thus not only bore the pressure from the contact surface but also received pressure from the support structure, enabling it to reach saturated adhesive force without requiring an excessively high pre-pressure [56]. Secondly, when the tilt angle of the upper pillar structure increased, the same pre-pressure generated a greater tangential force, which caused the adhesive structure to undergo slight sliding or shifting on the target surface. This adjustment helped the structure better adapt to the microgeometries of the target surface, reducing localized noncontact areas [57,58].

**Fig. 4.**
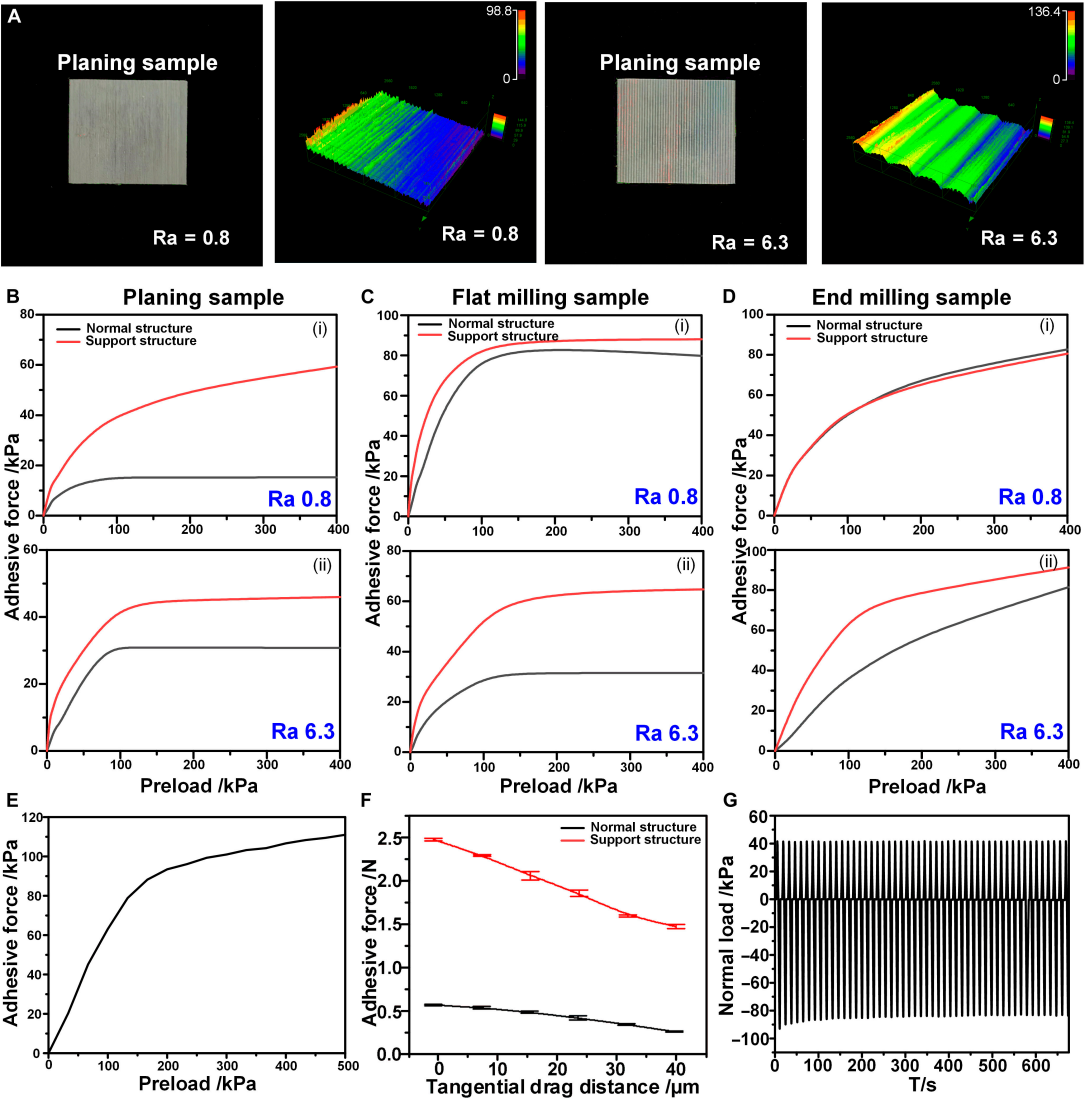
Adhesion performance of the intelligent bionic adhesive structure. (A) The planer-machined samples and their confocal images in the rough surface adhesion test; the other samples and confocal images are shown in Fig. S4. (B) The adhesion comparison results of the normal bionic dry adhesive structure and the hierarchical bionic dry adhesive structure for planer-machined roughness samples. (C) The adhesion force results of the normal bionic dry adhesive structure and the hierarchical bionic dry adhesive structure for roughness samples processed by a flat milling machine. (D) The adhesion comparison results of the normal bionic dry adhesive structure and the hierarchical bionic dry adhesive structure for vertical milling roughness samples. (E) The performance of the intelligent bionic adhesive structure on a smooth surface. (F) The drag distance versus the adhesion force for the normal bionic dry adhesive structure and the hierarchical bionic dry adhesive structure obtained in the load–drag–pull (LDP) test. (G) The cyclic test plot of the adhesion force of the hierarchical bionic dry adhesive structure.

After analyzing the simulation results regarding the adhesion performance of the hierarchical bionic dry adhesive structure on rough surfaces, this study designed and conducted a comparative load–pull (LP) test. This test was performed to evaluate the adhesion characteristics of the proposed structure on samples with different roughness levels. The goal was to characterize adhesion performance, assess the service life of adhered samples, and compare interfacial contact states. The standard roughness comparison samples, which are representative of common machined parts in engineering, were used to quantify the roughness using the roughness average (Ra) value, which reflected irregularities in microgeometry. By using laser confocal microscopy technology, the 3-dimensional morphology of an object’s surface could be accurately obtained, and the specific value of surface roughness was calculated by the supporting software. This value directly reflected the degree of roughness or smoothness of the surface. In this study, 3 machining methods, namely, vertical milling, flat milling, and planer, were employed to test the samples, and each of them contained 4 different roughness levels. Since the machined samples were too large to be placed directly on the probe end, a waterborne polyurethane acrylate (NOA71, commercially available, Nolan Corporation, USA), which was molded to the same shape but with thickness, volume, and mass much less than those of the machined samples, was used as a test surface. Figure 4A shows the planer machining samples with Ra = 0.8 μm and Ra = 6.3 μm and their confocal characterization. The other samples and their confocal features are described in Fig. S4.

The LP adhesion test method was used to compare the adhesion effects of hierarchical bionic dry adhesive structures and ordinary bionic dry adhesion junctions on samples of different roughnesses. Figure 4B shows the results of the planer method; at Ra = 0.8 μm, the normal structure achieved only 13 kPa, indicating nearly no adhesion, whereas that of the hierarchical structure reached 62 kPa. As the surface roughness increased, the adhesion effect slightly improved. However, at Ra = 6.3 μm, where the patterns became too wide and high for effective adhesion, there was a decrease in the force for both structures; namely, the hierarchical structure’s adhesion dropped to 48 kPa, and that of the normal structure remained at 26 kPa. In particular, the machining characteristics of the planing process resulted in a highly uneven surface, with distinct tool marks (the planing samples and their confocal features are described in Fig. S4B). Moreover, when the surface roughness was relatively low, not only did the cut lines undulate in height, but the spacing between the cut lines was smaller, leading to a reduced effective contact area during interfacial interaction. As a result, the adhesion performance of the normal adhesive structure decreased as the surface roughness of the sample decreased. As the surface roughness increased, the spacing between the cutter lines became wider, and the undulations of the cutter lines became less pronounced, thus slightly improving the adhesion properties. The flat milling results are displayed in Fig. 4C. At Ra = 0.8 μm, the hierarchical structure had an adhesion of 92 kPa, and the normal structure had an adhesion of 80 kPa. At Ra = 6.3 μm, the adhesion of the normal structure decreased dramatically to 30 kPa, which was too low for engineering applications; meanwhile, the proposed hierarchical structure still had an adhesion of 65 kPa. Figure 4D shows the adhesion test results for the end milling surfaces with different roughness levels. At Ra = 0.8 μm, the hierarchical adhesive structure achieved a force of 83 kPa, and the normal bionic dry adhesive structure reached a value of 80 kPa, both of which could be adequate for most work environments. However, as roughness increased to Ra = 6.3 μm, the hierarchical structure maintained stable adhesion performance at 95 kPa, whereas the normal structure showed a decline in adhesion performance. Thus, it could be concluded that the hierarchical bionic dry adhesive structure could adapt well to rough and complex surfaces. In addition, in some complex surface adhesion tests, the ordinary bionic dry adhesive structure almost completely lost the adhesion effect, whereas the hierarchical bionic dry adhesive structure still maintained a certain working ability. The comparison results of the adhesion effect of the 2 samples for different surface roughness values are presented in Fig. S5. As a control group, the experimental results depicting the adhesion force of the hierarchical bionic dry adhesive structure on smooth surfaces are displayed in Fig. 4E. The adhesion force of this structure on smooth surfaces was 111 kPa, which was notably higher than the adhesive force (typically ranging from 10 to 100 kPa) observed in most existing studies of gecko-inspired adhesion structures on smooth surfaces [23,29,32,46,59–63]. This comparison suggests that the introduction of the supporting layer not only preserved the adhesion performance of the smart structure on smooth surfaces, but enhanced it.

The hierarchical bionic dry adhesive structure demonstrated the ability to regulate interfaces contact and exhibited strong surface adaptability. To visualize this, we conducted optical microscopy observations. When the conventional bionic dry adhesive structure came into contact with a transparent, smooth glass surface featuring complex topography, the mushroom-shaped adhesion elements lost optimal contact under the more protruding regions of the glass. In contrast, the hierarchical bionic dry adhesive structure, supported by a tilted support layer, compensated for these surface irregularities. As a result, the adhesive structures with hierarchical designs maintained their normal morphology and contact state, thereby ensuring the proper functioning of adhesion. In addition to the function of morphology compensation, when the adhesion structure was subjected to force, it could function similarly to a spring, i.e., producing a restoring force, which helped the adhesion structure to more closely fit the target surface and enhanced the adhesion effect. Additionally, the investigation revealed a comparison between the adhesion forces of the hierarchical bionic dry adhesive structure and conventional adhesion structures on irregular rough and smooth surfaces. The experimental results demonstrated that the hierarchical bionic dry adhesive structure exhibited significantly better adhesion performance on both surface types compared to the conventional structure. On irregular rough surfaces, the hierarchical bionic dry adhesive structure adjusted the interfacial contact state, effectively compensating for morphological variations, thus reducing the impact of surface irregularities and achieving higher adhesion forces. On smooth surfaces, the introduction of a supporting layer enhanced the overall mechanical properties of the hierarchical bionic dry adhesive structure, leading to increased adhesion forces. Experimental images and their principles are described in Fig. S6.

This study performed load–drag–pull tests for the common bionic dry adhesive structure and its hierarchical variants to evaluate their adhesion stability under different conditions. The experimental results showed that when the preset pressure (*F*_pre_) was set to 0.5 N and the drag distance (*l*_d_) was increased to 40 μm, the desorption force (*F*_p_) of the common bionic dry adhesion decreased by 55%, as illustrated in Fig. 4F. In contrast to this structure, the hierarchical bionic dry adhesive structure showed superior performance under the same test conditions; namely, when the drag distance reached 40 μm, the desorption force of the hierarchical bionic dry adhesion stayed at 1.7 N, which was only 32% lower than that of the initial 2.5 N when there was no drag (i.e., *l*_d_ = 0). Therefore, it could be concluded that when the drag distance increased, the decrease in the adhesive force of the hierarchical bionic dry adhesive structure was moderate, particularly the decrease in the *F*_p_ value. The hierarchical bionic dry adhesive structure demonstrated excellent adhesion stability, effectively addressing challenges related to adhesion degradation that could lead to peeling. Finally, this reliability demonstrated its suitability for practical applications.

In the performance analysis of hierarchical bionic dry adhesive structures, in addition to the key indicator of adhesion effect, the service life should also be examined. It should be noted that the feasibility of engineering applications is highly dependent on the stability and durability of the product in repeated use. Considering that the samples will undergo hundreds of adhesion–desorption cycles in application scenarios, cyclic adhesion tests were conducted in this study to assess their stable performance after multiple uses. As shown in Fig. 4G, a total of 50 adhesion–desorption cycles were performed. At the end of the cycle test, the maximum adhesion of the hierarchical bionic dry adhesive structure was 84 kPa, which was only a slight decrease of 8.7% from the 92 kPa at the beginning of the cycle, indicating the stability of the structure under continuous use. Next, after the sample was left for a month, a second 50-adhesion–desorption-cycle test was conducted, which showed that the final maximum adhesion remained at 84 kPa, a decrease of only 3.4% from the 87 kPa at the beginning of the cycle. The maximum adhesion values measured at the end of the 2 cycles were almost identical, which proved the stability of the hierarchical bionic dry adhesive structure after several uses. The results of the second adhesion–desorption cycle test are shown in Fig. S7.

**Fig. 5.**
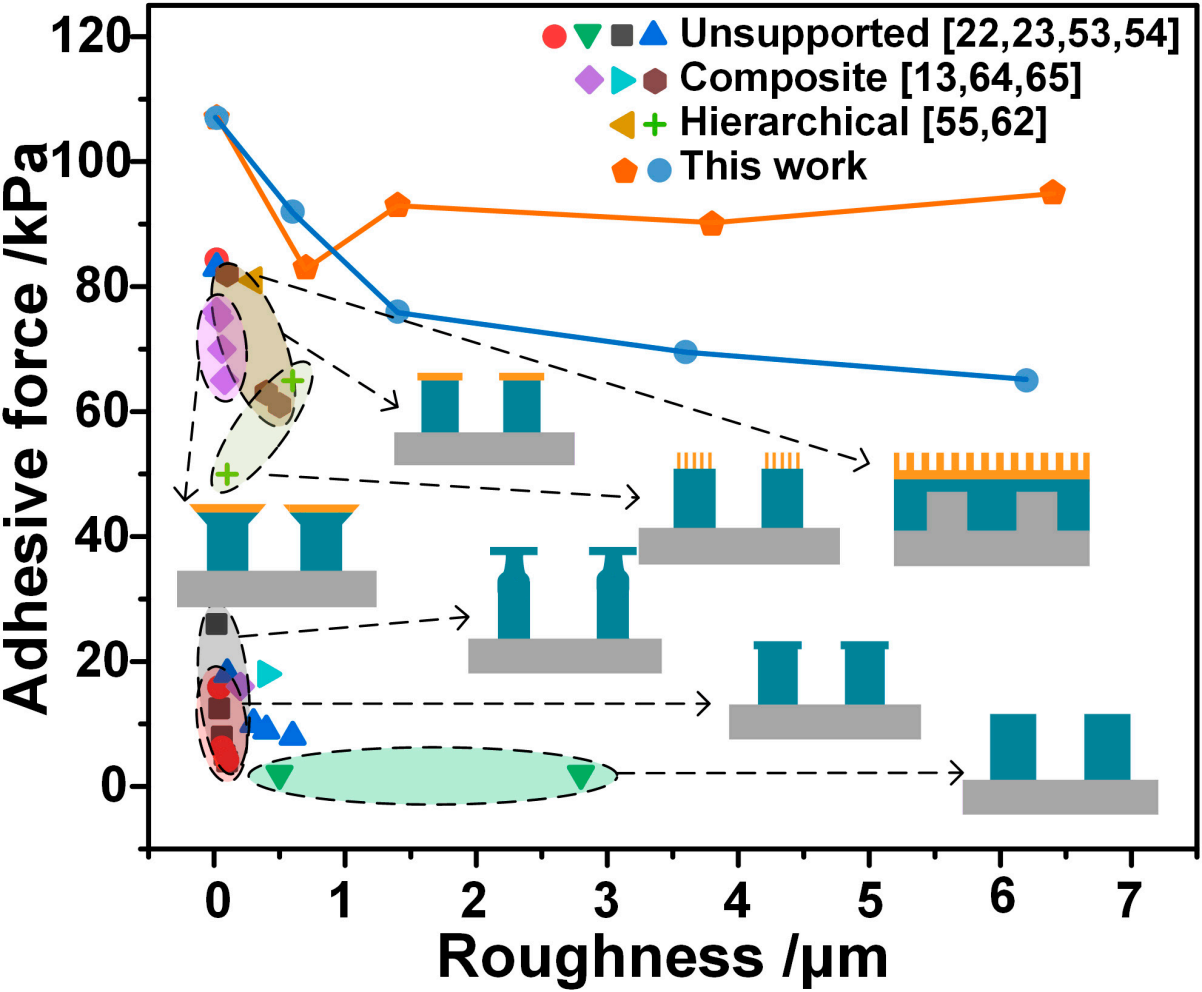
Comparative roughness–adhesion plots of unsupported structures, composite structures, other hierarchical structures, and this work.

To illustrate the superior adhesion performance of the hierarchical bionic dry adhesive structure on rough surfaces, this paper compared the adhesion performance of the hierarchical bionic dry adhesive structure on vertical and flat milled samples, with the adhesion performance of other typical adhesive structures, including unsupported structures, composite structures, and hierarchical structures, as shown in Fig. 5 [13,22,23,53–55,62,64,65]. Most unsupported structures are conventional bionic adhesive structures with low adhesion toward rough surfaces. The introduction of hierarchical and composite structures caused an increase in the adaptability of the bionic dry adhesive structures toward rough surfaces. Comparisons here show the adhesion force of the hierarchical bionic dry adhesive structure in this paper on the end milling samples (orange line) and flat milling samples (blue line). In addition, to evaluate the differences in adhesion performance between the soft substrate adhesive structure and the hierarchical bionic dry adhesive structure on these surfaces, a series of adhesive structure samples were designed and fabricated, incorporating a sponge (50 ppi) as the backing material and a mushroom-shaped adhesive structure as the adhesive layer (Fig. S8) [66]. The adhesion performances of both structures were compared using the LP test method on 2 different rough surfaces: one was a polylactic acid plate with irregular roughness (Ra = 3.79 μm), and the other was a polytetrafluoroethylene plate with a regular sinusoidal rough surface (Ra = 4.738 μm). The experimental results demonstrated that the hierarchical bionic adhesive structure outperformed the soft substrate adhesive structure on both surfaces. Notably, on the rough surface with regular ripple patterns, the adhesion strength of the hierarchical bionic dry adhesive structure was 201.62% higher than that of the sponge backing adhesive structure. This enhancement was attributed to the smaller dimensions of the micropillars in the pillar layer of the hierarchical adhesive structure, which allowed for better morphological compensation and resulted in a larger contact area. In contrast, while the sponge substrate, due to its high compressibility and softness, could adapt to the irregularities of the surface to some extent, its flexibility prevented it from conforming closely to the complex surface with continuous variations in height. Therefore, the micropillars in the hierarchical bionic dry adhesive structure, with their smaller scale, could better accommodate the surface morphology changes. This design not only increased the contact area with the surface but also improved the morphological compensation capability on complex surfaces, thereby enhancing the adhesion strength. Detailed experimental data and analysis can be found in Fig. S9.

The comparison results showed that the hierarchical bionic dry adhesive structures in this paper could be used with rough surfaces from the nanometer to the micrometer scale and could achieve stable adhesion performance.

The nervous system helps geckos realize the gecko paw’s perception of the surrounding environment. Learning about the nervous system in bionic engineering is the sensing technology. The hierarchical dry adhesive structure features a support area with 2 surfaces and a gap, resembling capacitive sensors. Therefore, by modifying this structure to incorporate capacitive sensor elements, its adaptability and adhesion can be retained while adding sensing capabilities. Unlike most other adhesive structures with sensing functions, the intelligent adhesive structure in this paper was designed to produce a structure that integrates both adhesive functions and sensing functions without adding a separate sensing system to the adhesive structure. The sensing structure not only detected the interface contact and provides feedback on the contact interface morphology but also enhanced the adhesion performance on rough surfaces as part of the adhesive structure. Because of its integrated characteristics, the sample has higher mechanical strength and flexibility. For example, its size can be changed in practice according to the usage scenario. They are also more environmentally stable than assembled sensors (e.g., a sensor made of gel material).

**Fig. 6.**
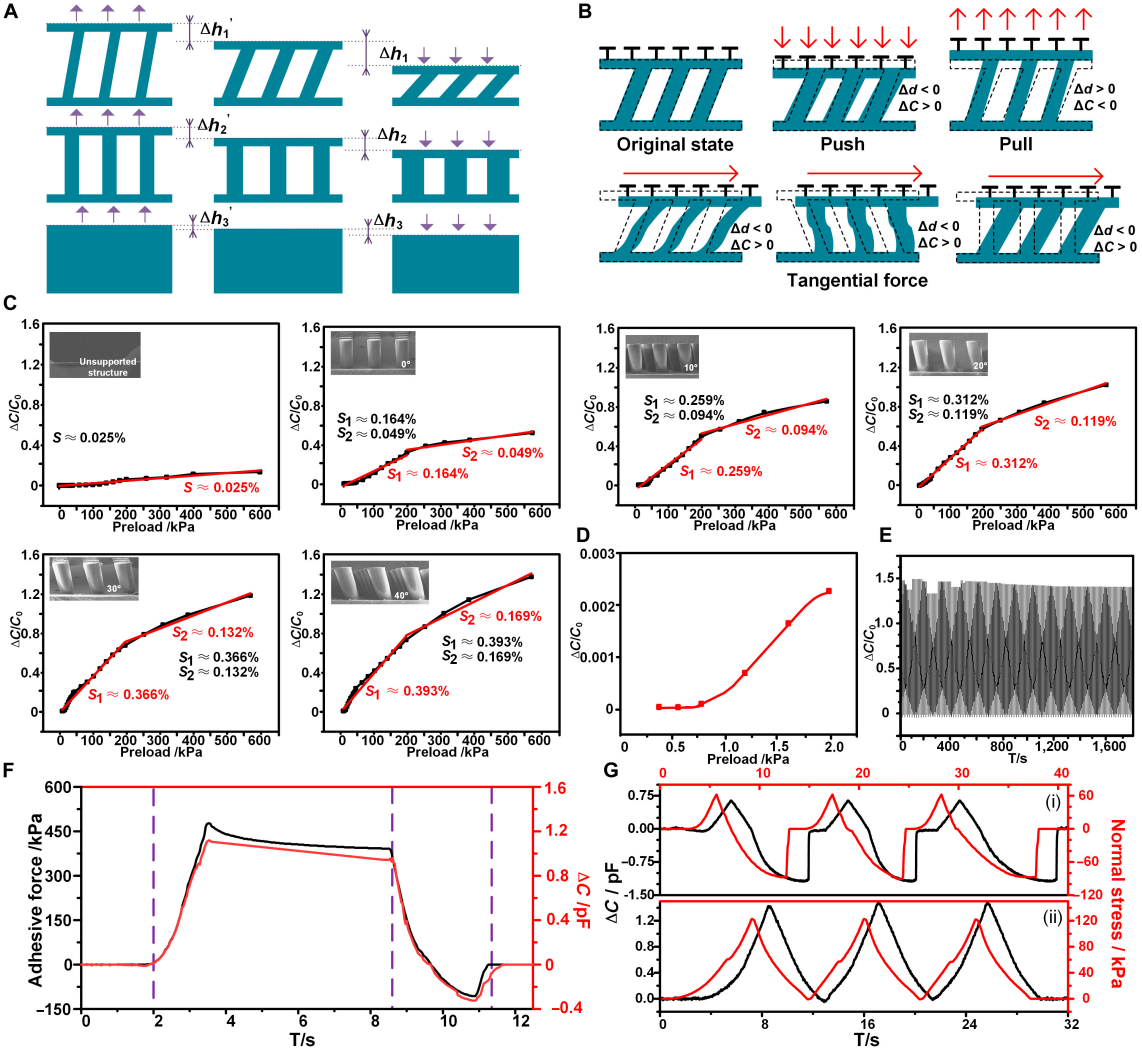
Sensing properties of the intelligent bionic adhesive structure. (A) The comparison results of the deformation degree of the inclined supported, vertically supported, and unsupported structures under external forces. (B) The changing trends of the bipolar plate spacing and the resulting capacitance of the hierarchical bionic dry adhesive structure under pressure, tension, and horizontal force. (C) The sensitivity comparison graph of the support structure at different tilt angles. (D) The resolution of the intelligent adhesive structure. (E) The cyclic test plot of the sensing function of the intelligent adhesive structure. (F) The capacitance changes at different stages of the press–pull–desorption test. (G) The plot of capacitance signal changes during 3 cycles of the press–pull–desorption (upper) and press–release (lower) processes.

The composition of the integrated adhesion and sensing structure from top to bottom is as follows: an ordinary bionic dry adhesive structure layer, a flexible electrode material, an inclined support structure, a flexible electrode material, and a polydimethylsiloxane (PDMS) substrate. The inclined cylindrical support structure, in addition to improving surface adaptability, also supports the application of a pressure-based capacitive sensor. As shown in Fig. 6A, under the same initial height and pre-pressure application, the inclined support structure exhibited superior compression performance compared to the vertical support structure and the unsupported structure. This superior compression performance indicated not only that the sensor could more effectively capture small deformation signals but also that it could maintain higher measurement stability and reliability in complex and variable external environments or under extreme operational conditions. Therefore, the application of the tilting support structure could not only broaden the testing range of capacitive flexible sensors but also significantly improve their measurement accuracy and applicable scenarios.

The change in the capacitance value was related to the distance between the upper and lower polar plates and was inversely proportional to it. Therefore, when loads were applied to the structure from different directions, the inclined support structure was deformed in different ways, including compression, elongation, or bending deformations, which directly affected the distance between the upper and lower polar plates (Δ*d*), thus further influencing the capacitance value (Δ*C*), as shown in Fig. 6B. When the sensor was subjected to a certain pressure or a tangential shear force, the tilted support structure underwent compressive deformation, causing the distance (*d*) between the 2 polar plates to decrease (Δ*d* < 0). Due to the relationship between the distance and the capacitance value, the capacitance value (*C*) increased (Δ*C* > 0). In contrast, when the sensor was subjected to a tensile force, the support structure elongated or bent and deformed, resulting in an increase in the distance between the 2 polar plates (Δ*d* > 0), and accordingly, the capacitance value decreased (Δ*C* < 0).

As displayed in Fig. 6A, the tilt angle of the support structure affected the distance between the 2 plates under the external force’s effect. To evaluate this influence on the sensing performance, this study tested the capacitive sensors for different support angles, using the dimensionless capacitance change rate Δ*C*/*C*_0_ to reflect detection capability. The sensitivity value (*S*), which represents a key metric for evaluating sensor performance, varied with the tilt angle of the support structure. Figure 6C shows the sensitivity calculation results for support structures at different angles. Sensitivity was higher below 200-kPa compressive stress and lower above that threshold, necessitating a division into 2 segments. For the unsupported structure, the sensor’s sensitivity value was lower, and it was almost impossible to measure the change in the stress value. This was mainly because the dielectric of the whole block had a small modulus of elasticity, and the deformation during the compression process was too small, resulting in a small change in the capacitance value and less feedback. The sensitivity calculation results of the vertical support structure were as follows: the sensitivity of the first section (0 to 200 kPa) was 0.164% kPa^−1^, which was greater than that of the second section of 0.041% kPa^−1^. The sensitivity of the integrated structure at a support angle of 10° was calculated to be 0.259% kPa^−1^ at the first stage, which was 1.58 times higher than that of the vertically supported integrated structure, and 0.094% kPa^−1^ at the second stage, which was 2.29 times higher than that of the vertically supported integrated structure at the second stage. The sensitivity of the integrated structure at a support angle of 20° was 0.312% kPa^−1^ and 0.126% kPa^−1^ for the first and second segments, respectively. The sensitivity of the integrated structure with a support structure inclination angle of 30° was calculated to be 0.366% kPa^−1^ and 0.132% kPa^−1^ for the first and second segments, respectively. The sensitivity of the integrated structure at a support structure inclination angle of 40° was 0.428% kPa^−1^ and 0.194% kPa^−1^ for the first and second segments, respectively, which were 2.61 and 4.73 times higher than those of the vertically supported integrated structure, respectively. The experimental comparison results indicated that both the first and the second sections of the integrated structure achieved the best sensitivity performance at an inclined support angle of 40°. Therefore, the tilt angle of the support structure in the integrated adhesion or sensing structure was finally set to 40°.

The minimum pressure resolution of a flexible capacitive pressure sensor represents a measure of its ability to sense and convert the smallest amount of force change into a detectable signal during the operation process. To quantify this measure, this study tested the minimum resolution of the intelligent adhesive structure. As shown in Fig. 6D, during the test, there was a change in the capacitance change rate when the normal compressive stress gradually increased. Namely, when the compressive stress was increased by 0.2 kPa (i.e., approximately 0.02 N), the magnitude of the change in the capacitance change rate was small, making it challenging to be recognized or distinguished accurately. However, when the compressive stress was increased by 0.4 kPa (i.e., approximately 0.04 N), the capacitance change rate showed notable fluctuations. Thus, it could be concluded that the minimum force that could be detected by the intelligent adhesive structure was 0.04 N.

Further, stability is an important characteristic that indicates whether the output value of a sensor can be maintained within a predetermined and acceptable range of fluctuations under the condition of continuous, repeated use. In view of this, this study conducted normal stress cycling tests on intelligent adhesive structures to evaluate their stability performance over extended periods of use. The 200-cycle-test data plot is shown in Fig. 6E, where it can be seen that the capacitance change rate exhibited small fluctuations without any marked offset, drift, or other instability when the normal stress was constant. This stable performance indicated that the integrated adhesion or sensing structure could maintain the coherence and consistency of its sensing performance over multiple test cycles, independent of the number of tests or time extension. Therefore, it could be concluded that the sensing performance of the intelligent adhesive structure was stable.

The test process performed in the experiment to explore the relationship between the adhesion force and the capacitance value is shown in Fig. 6F. The results demonstrated that there was a certain degree of consistency between the adhesion force and the capacitance value, which was indicated by a parallel development trend of the adhesion force curve and capacitance change curve with time. During the experiment, the capacitance change rate also increased gradually when the external pressure was gradually applied, but it gradually stabilized when the pressure was constant. After that, when the tensile force was applied in the reverse direction, the sample and the contact interface affected the adhesion force, and the capacitance change rate was negative; with the increase in the adhesion force, the value of the capacitance change rate decreased, but with the decrease in the adhesion force, the value of the capacitance change rate increased. The capacitance change rate tended to stabilize when it was completely desorbed.

The close correlation between the adhesion force and the capacitance change could be an effective way to monitor the interfacial contact state in real time. Sub-image (i) of Fig. 6G illustrates the results of 3 rounds of the repetitive monitoring of the adhesion–desorption process of the intelligent adhesion system. At the stage of the pre-pressure application, the capacitance change rate had a positive value and increased gradually with the pressure value. Subsequently, when the pull-off force was applied, the capacitance change rate decreased along with the decrease in the adhesion force, which indicated that the sample was in the state of gradual deadhesion. Until the complete deadhesion, the capacitance change rate went back to the initial value and tended to stabilize, reflecting the completion of the deadhesion process. Sub-image (ii) of Fig. 6G shows 3 rounds of the repetitive monitoring process of the intelligent adhesive structure under the 0–120 kPa–0 pressure change. When pressure was applied, the capacitance change rate increased significantly; but when pressure was removed, the capacitance change rate decreased accordingly. The experimental results also showed that the adhesion state of the intelligent adhesive structure could be monitored in real time by accurately collecting and analyzing the capacitance data. This could not only quantify the pre-pressure magnitude to determine the occurrence of dislodgement events accurately but also provide rapid feedback under external environmental disturbances to ensure continuous monitoring and evaluation of the adhesion state.

**Fig. 7.**
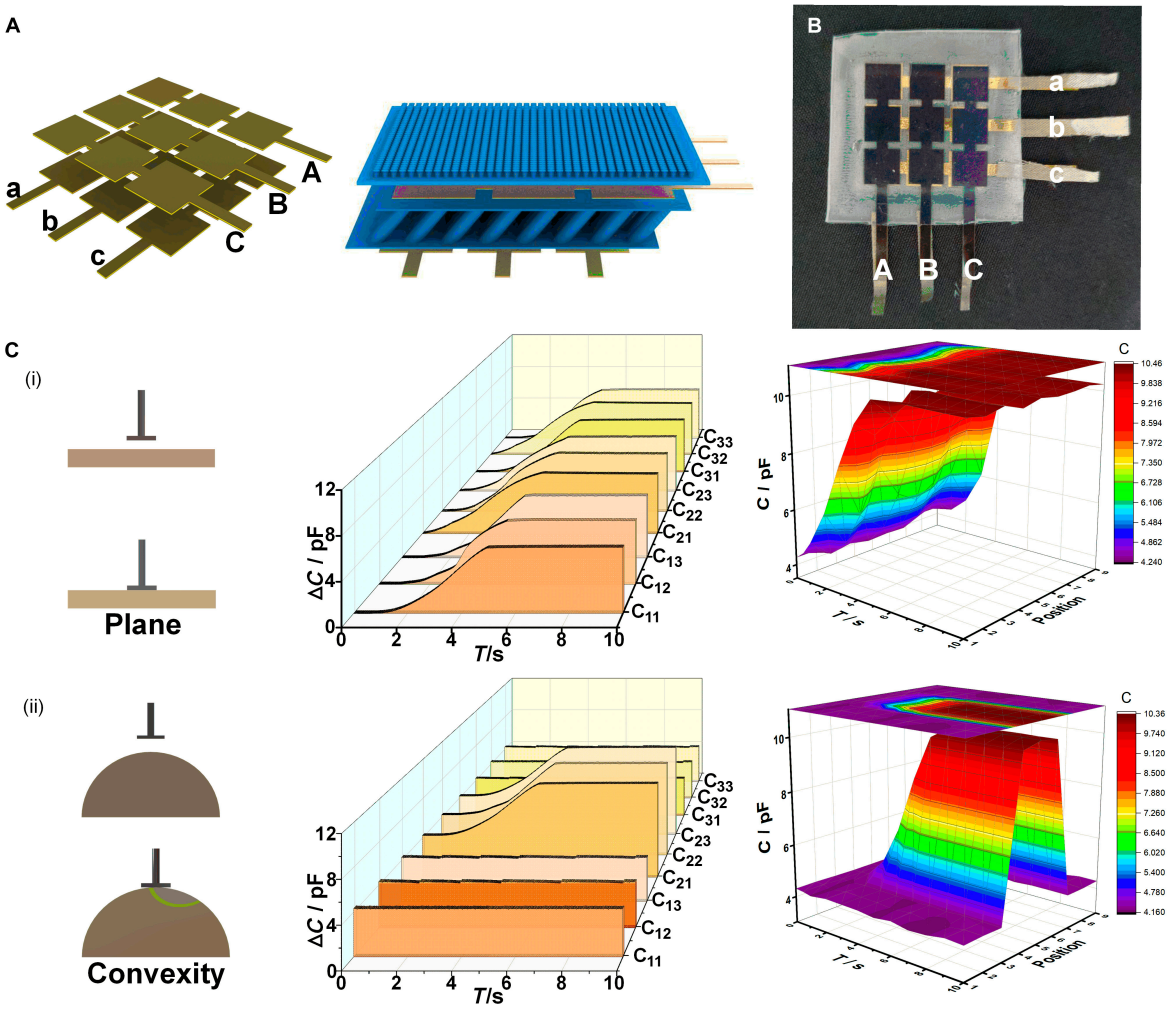
Regionalized intelligent bionic adhesive structure. (A) The 3 × 3 arrayed electrodes constructed by the cross-linking method and bonded to each end of the support layer of the intelligent adhesive structure. (B) The physical diagram of the regionalized intelligent adhesive structure. (C) The interfacial sensing tests of the regionalized intelligent adhesive structure with the planar and convex probes and detection of capacitance changes for each region of the 3 × 3 array.

The intelligent adhesive structure that covered the entire block could not effectively detect the target surface’s shape. Inspired by biological nervous systems, this study constructed a regionalized design using flexible array sensors. As illustrated in Fig. 7A, a hierarchical composite structure with a cross-linking approach allowed converting larger electrodes into a 3 × 3 array of unit sensors. The arrayed electrodes were aligned with each other, and the area directly opposite to the 2 upper and lower squares denotes the detection area; the resulting physical object is shown in Fig. 7B. Similarly, in addition to preparing 3 × 3 arrays, it was also possible to prepare N × N matrices, and the intelligent adhesive structure of a 3 × 3 matrix was used as an example for the preparation, testing, and characterization processes. Marking each derived lead separately, 9 signal values (C_11_ to C_33_) were obtained, and each individual array was labeled with a name for recording experimental data. The detailed array names can be found in Table S4.

As shown in Fig. 7C, the test was conducted using applied pressure, with a stretching machine controlling the probe to approach the adherent sample gradually while continuously applying a fixed pressure. During this process, a capacitive signal collector recorded signals from a 3 × 3 array of points. When the contact interface between the probe and the sample was planar, the capacitance values of the 9 points changed almost simultaneously, and the difference between their measured capacitance values is small, which could be used to provide feedback on the normal force situation based on the capacitance value. The crosstalk situation was detected next using the end of the probe with protrusions for the pressure test. Due to the protruding end of the probe, only the middle 3 points experienced stress in the normal direction, which resulted in detectable changes in these 3 capacitance signals, whereas the remaining 6 points showed no notable changes. The reason that the array-sensor signals in the uncontacted region did not change is that each individual array was surrounded by PDMS as a support layer, and the underlying support structure did not undergo large morphological mutations to compensate for the change in morphology. Based on the intelligent adhesive structure, an array form was introduced, and adhesion experiments were conducted on actual objects to detect different morphological features. The array sensors generated different signal curves, which could be used to retrieve the interfacial morphological features of the target objects through postprocessing. The physical testing of interfacial shape sensing of regionalized intelligent bionic dry adhesive structures are shown in Fig. S10.

## Conclusion

Inspired by the climbing ability of a gecko’s paws in complex environments, this study proposes an intelligent adhesive structure and introduces its preparation method. In addition, based on the principle of fracture mechanics, a cohesion model is constructed and verified by experimental tests. The experimental results confirm the superiority of the hierarchical bionic adhesive structure in rough surface adhesion. By combining the hierarchical bionic dry adhesive structure with flexible capacitive sensor technology, the adhesive structure is endowed with the real-time sensing ability for the adhesion effect without affecting the adhesion effect on a rough surface. The flexible electrodes are arrayed to develop a regionalized intelligent adhesive structure, which realizes a sharp perception of the contact interface morphology through the analysis of the adhesion and sensing performances and the interface morphology sensing effect. In terms of the requirement for real-time monitoring of the high adhesion to rough surfaces and the shedding and adhesion effects in practical applications, the proposed intelligent adhesive structure could be used in future dry adhesion applications requiring precise and efficient adhesion and sensing.

## Methods

### Materials

Unless stated otherwise, solvents and chemicals were obtained commercially and used without further purification. The PDMS prepolymer was purchased from Dow Corning, USA. Photoresist No. 2050 from the negative SU8 series was purchased from Suzhou CChip Scientific Instrument Co., Ltd. The polyimide (PI) film was purchased from Wuxi Shunxuan New Material Co., Ltd. NOA71 was commercially available from Nolan Corporation, USA. The polyurethane sponge was commercially purchased from Hangmei Company, China.

### Preparation of intelligent adhesive structure samples

A reverse-type mushroom-shaped glass hard-substrate mold was created by double-sided exposure lithography and then filled with a 10:1 mixture of the PDMS prepolymer (Axson ESSIL 296) and curing agent. The mushroom-type flip mold made of polystyrene was filled with a 10:1 mixture of the PDMS prepolymer and curing agent, and a vacuum was applied to fill the pores and evacuate the air bubbles. Then, a screeding machine was used to screed the molds at 5,000 rpm.

Counter-type cavities with tilted pillars were made by photolithography, creating a flexible mold using a 10:1 mixture of the PDMS prepolymer and curing agent. The mask had a diameter of 60 μm and a pitch of 120 μm. Photoresist No. 2050 from the negative SU8 series was spin-coated on clean glass at 500 r/min for 10 s and then at 1,500 r/min for 30 s. Then, this was followed by a step baking process at 65 °C for 5 min and at 95 °C for 22 min on a 40° inclined table and exposure to ultraviolet light at 365 nm for 25 s. After exposure, the adhesive was baked at 65 °C for 5 min and then at 95 °C for 10 min, with 11 min of development by SU-8 Developer.

Before bonding, photoresist molds were pre-cured at 80 °C. The proposed intelligent adhesive structure was created by sputtering Cr (40 nm), Au (80 nm), and SiO_2_ (100 nm) sequentially on a 30-μm PI film, with an additional SiO_2_ layer on the opposite side. The PDMS was filled, evacuated, and spin-coated at 5,000 rpm for 40 s. The flexible motor film was cationized and pressed onto the uncured PDMS, using rolling for thickness uniformity. The process was repeated for the top structure. For the intelligent adhesive structure, the metal-treated PI film was laser cut, and the electrode material was attached before curing the PDMS. After curing, another PDMS layer was added to bond the components, and electrodes were connected using leads.

### Preparation of NOA samples with the same roughness as the processed samples

The PDMS material was used to create a counter-mold that replicated the surface of a machined standard roughness comparison sample. Next, the NOA material was poured into this counter-mold through mold turning. The results of the NOA test sample closely matched the properties of the original machined surface. Consequently, the surface shape of the NOA sample was consistent with that of the custom-made machined standard roughness comparison sample.

### Preparation of sponge backing adhesive structure samples

Mushroom-type flip molds made of polystyrene were filled with a 10:1 mixture of PDMS prepolymer and hardener and vacuumed to fill pores and expel air bubbles. The molds were then homogenized with a homogenizer at 5,000 rpm. The mold was placed on a 90 °C baking table for pre-curing, and the sponge (50 ppi) was placed on top of the PDMS. After waiting for curing, the mold was demolded and cut to the appropriate size.

## Data Availability

Data are available on request from the authors.

## Supplementary Materials

Supplementary 1 Sections S1 to S11 Figs. S1 to S10 Tables S1 to S4 Movies S1 and S2
