# Supplementary material for: Gecko-Inspired Intelligent Adhesive Structures for Rough Surfaces
Source: Research (Wash D C). 2025 Feb 25;8:0630. doi: 10.34133/research.0630 (PMC11850978; doi:10.34133/research.0630)
Supplement: Supplementary 1 — Sections S1 to S11 Figs. S1 to S10 Tables S1 to S4 Movies S1 and S2 [file research.0630.f1.zip › Supplementary materials.docx]

**SUPPLEMENTARY MATERIALS**

**Gecko-inspired Intelligent Adhesive Structures for Rough Surfaces**

Yawen Shao, Miao Li, Hongmiao Tian*, Fabo Zhao, Jian Xu, Hongrong Hou, Zhijun Zhang, Duorui Wang, Xiaoliang Chen, Wenjun Li, Hongjian Yan, Jinyou Shao

Section S1. Roughness Information for the Frosted Glass.

In verifying the adhesion effect of the hierarchical bionic dry adhesive structure when confronted with objects with different surface features, the frosted glass used and its confocal features are shown in Fig.S1. The Arithmetic mean deviation of the actual profile of the hair glass was obtained by confocal microscopy measurements as 1.753 μm. And the maximum height of actual profile was 28.482 μm.


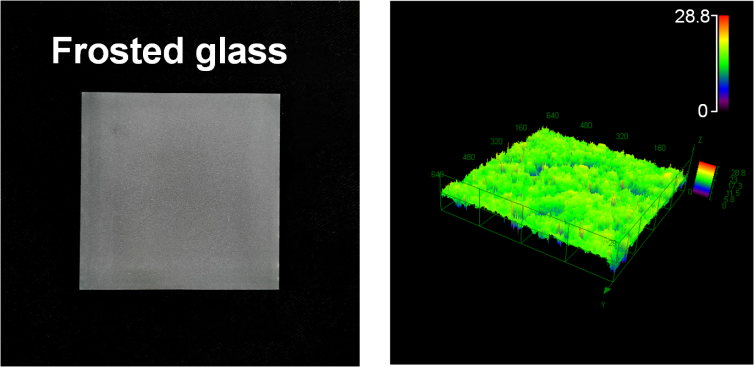


Fig.S1 The frosted glass sample and its roughness characterization.

Section S2. Cohesion modeling and Interface Fracture Damage Criteria.

To investigate the interface contact law of multilevel structures, the corresponding physical mechanics model should be established. It was based on the cohesion model and the interface fracture damage criterion first. The simulation software used in this paper is the ABAQUS. In the simulation test model of this paper, only the normal displacement motion of the adherent structure surface and the target surface was simulated. Under the action of normal adhesive force, the crack caused by the separation process was an open crack (**Fig. S2A**), so the simulation process was based on open crack for modeling analysis and discussion.

When modeling crack extension based on linear elastic mechanics in finite element simulation, the crack needed to be predefined in the sample. Due to the singularity in the finite element simulation of the crack tip, scientists have proposed the concept of cohesion modeling. The cohesion was expressed as a function of the crack tensile displacement, denoting the small distance between the upper and lower surfaces at the crack tip as the tensile displacement *δ*, then the stress *σ* at the interface there is an equation of *δ* (equation S1):

$\text{σ }\text{=}\text{ }\text{f }\text{(}\text{δ }\text{)}$ (S1)

where *σ* is the tension on the surface at the crack tip (N)；*δ i*s the displacement of interfacial tension at the crack tip (m). The process of crack unfolding will form a new crack fracture surface, the energy released by this process, we call the fracture energy (*G*), and its calculation formula is as follows (equation S2):

$\text{G}\text{ = ∫}\text{σ}\text{ d}\text{δ}\text{ = ∫f }\text{(}\text{δ}\text{ }\text{)}\text{ d}\text{δ}$ (S2)

When the cohesive region was subjected to load, the value of the stress at the material point will increase with the crack expansion and when the stress *σ* reached a maximum value of *σ*_max_, then the material point stress was subjected to the load reaches its maximum value. Beyond the maximum value, damage begins to occur, and at this time the stress at this point will gradually decline until it reached zero, and this stage was called the damage evolution decline stage of the material. The cohesion model introduces the cohesive region and cohesive force, which avoided the trouble of the stress singularity at the crack tip in the linear elastic mechanical analysis, and could simulate the mechanical behaviour of crack extension and the interface mechanical behaviour of composite materials more perfectly.

Since the separation process of dry adhesion could be regarded as the interfacial separation process of the composite material at the time, the classical bilinear eigenstructure law (**Fig.S2B**) was used in this chapter to characterize the separation process of the linear elastic body interface and the target surface. The cohesive zone model is formulated as follows (equation S3):

$\text{F}\text{=}\left\{ \begin{matrix} \text{F}_{\text{n}} \\ \text{F}_{\text{s}} \\ \text{F}_{\text{t}} \end{matrix} \right\}\text{=}\text{A}\left[ \begin{matrix} \text{K}_{\text{nn}} & \text{K}_{\text{ns}} & \text{K}_{\text{nt}} \\ \text{K}_{\text{ns}} & \text{K}_{\text{ss}} & \text{K}_{\text{st}} \\ \text{K}_{\text{nt}} & \text{K}_{\text{st}} & \text{K}_{\text{tt}} \end{matrix} \right]\left\{ \begin{matrix} \text{δ}_{\text{n}} \\ \text{δ}_{\text{s}} \\ \text{δ}_{\text{t}} \end{matrix} \right\}\text{=}\text{A}\text{Kδ}$ (S3)

where *F* is the sum of interfacial separation forces; *F_n_* is the normal separation force exerted at the interface during interfacial separation(N); *F_s_* and *F_t_* are the tangential separation forces in both directions on the interface during interfacial separation(N); A is the total contact area of the interface(m^2^); *K* is the interfacial stiffness matrix (N·m^-2^); *δ*_n_, *δ*_s_ and *δ*_t_ are the tensor displacement of cohesive interfaces in three directions(m).

The stiffness of the interface starts to degrade and become smaller when the interface stress reached the maximum value (i.e., point b in **Fig.S2B**), and the initial damage judgment criterion was defined to determine when the interface stress reaches the maximum value. The initial damage judgment criterion used in this paper is the maximum nominal stress damage criterion (equation S4):

$\text{Max}\left\{ \frac{\text{σ}_{\text{n}}}{\text{σ}_{\text{N,max}}}\text{,}\frac{\text{σ}_{\text{s}}}{\text{σ}_{\text{S,max}}}\text{,}\frac{\text{σ}_{\text{t}}}{\text{σ}_{\text{T,max}}} \right\}\text{=1}$ (S4)

where *σ*_n_, *σ*_s_ and *σ*_t_ are the stresses on cohesive interfaces in three directions (MPa). The damage evolution law was the rate at which the parameter of the internal stiffness of the material decreases with increasing separation displacement, when the interfacial behavior caused the material to reach the initial damage criterion, it could be determined from both energy and displacement. In terms of displacement, the damage evolution was determined by the displacement of the interface opening at the failure site. When the stress reduction was zero, the separation displacement corresponding to point d in **Fig.S2B** defined that the separation has been completed.

Since this paper is to consider the pre-pressure and solve the problem of different surface contact, and based on the Van der Waals force to establish the model, the thickness of the adhesive layer can be ignored. Therefore, we chose to use the cohesive surface for modeling and analysis.


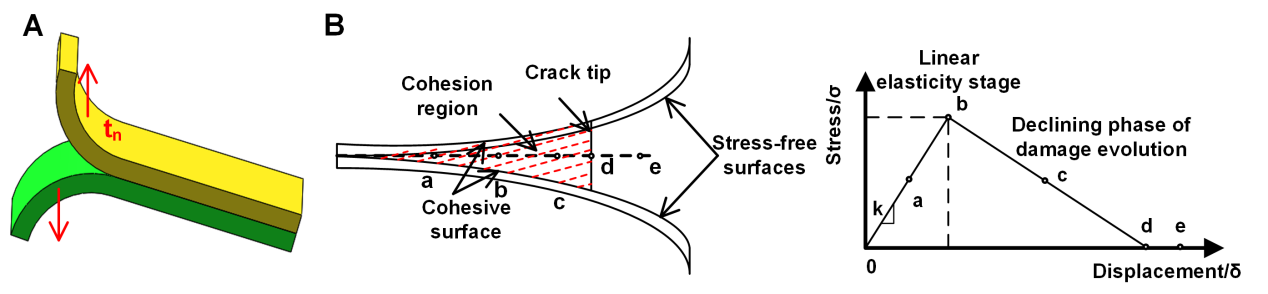


Fig.S2 The process of modeling the contact mechanics of interfaces with hierarchical bionic dry adhesive structures. (A) open cracks, where both surfaces of the crack will carry out along the direction normal to the crack surface, usually due to positive stresses acting on the object perpendicular to the crack plane. (B) bilinear intrinsic method, the process of separation of the linear elastic body interface and the target surface and its stress changes.

Section S3. Simulation modeling of the adhesion process of hierarchical bionic dry adhesive structures and ordinary bionic dry adhesive structures to rough surfaces.

The purpose of the simulation was to verify that the hierarchical structure can be better adapted to adhering to the target surface than the normal structure, and therefore the focus should be on the difference in adhesion ability with comparing the hierarchical structure with the normal structure. The modeling process of the physical model of planar adhesion, which was used as a basis for the modeling, is presented here.

The model for investigating the support structure's dimensions consisted of two main parts: the sample and the probe, as shown in **Fig.S3A**, with region a representing the support structure. The photoresist thickness was controlled at 17 μm, with the top mushroom structure also being 17 μm and a 5 μm connection to the tilted support, resulting in *h*_1_ = 0.022 mm and *h*_2_ = 0.12 mm for the tilted support. As other dimensions (*h*_3_, *l*_1_, *l*_2_) did not affect adhesion, here they were set as *h*_3_ = 1 mm, *l*_1_ = 16 mm, *l*_2_ = 20 mm. The glass probe was modeled as linear-elastic with Young's modulus *E* = 55 GPa and Poisson's ratio *γ* = 0.25, while the sample used a hyperelastic Neo-Hookean model with *E* = 2 MPa and *γ* = 0.46. When setting the parameters of the Neo-Hookean model in Abaqus, Young's modulus and Poisson's ratio could not be input directly, and they need to be obtained by calculation, in which the energy formula of the Neo-Hookean model is equation S5:

$\left\{ \begin{matrix} \text{W}\text{ = }\text{C}_{\text{10}} ({\bar{\text{ }\text{I}}}_{\text{1}} - 3 ) + \frac{\text{1}}{\text{D}_{\text{1}}} {\text{( }\text{J}\text{ - 1 )}}^{\text{2}} \\ \text{C}_{\text{10}}\text{ = }\frac{\text{μ}}{\text{2}} \\ \text{D}_{\text{1}}\text{ = }\frac{\text{2}}{\text{κ}} \end{matrix} \right.$ (S5)

where *C*_10_ and *D*_1_ are the parameters that should be entered in Abaqus. ${\bar{\text{I}}}_{\text{1}}$ is the first strain bias invariant. *J* is the correlation covariate. *μ* is the shear modulus and *κ* is the bulk modulus (equation S6).

$\left\{ \begin{matrix} \text{μ}\text{ = }\frac{\text{E}}{\text{2 }\left( \text{ 1 + }\text{ν}\text{ } \right)} \\ \text{κ}\text{ = }\frac{\text{E}}{\text{3 }\left( \text{ 1 - 2}\text{ν}\text{ } \right)} \end{matrix} \right.$ (S6)

Associative equations S5 and S6 can be obtained (equation S7):

$\left\{ \begin{matrix} \text{C}_{\text{10}}\text{ = }\frac{\text{E}}{\text{4 }\left( \text{ 1 + }\text{ν } \right)} \\ \text{D}_{\text{1}}\text{ = }\frac{\text{6 }\left( \text{ 1 - 2}\text{ν } \right)}{\text{E}} \end{matrix} \right.$ (S7)

Therefore, Young's modulus (*E* = 2 MPa) and Poisson's ratio (*γ* = 0.46), *C*_10_ = 0.34 and *D*_1_ = 0.24 should be entered in the material parameter interface.

The adhesion behavior between the adhesive sample and the glass probe was governed by cohesive contact properties, including interface adhesion (initial stiffness) and damage evolution (maximum traction, critical fracture energy) in the Interaction module. This study used a maximum nominal stress criterion, with damage parameters set to 0.45 in all directions, stiffness at 5000 N/mm, energy evolution type, B-K energy criterion for damage, and a critical fracture energy of 1E-5. To enhance convergence, the adhesion coefficient was set to 1E-9. In the ABAQUS/Standard contact analysis, the glass probe's lower surface acted as the master surface, while the PDMS film's upper surface was the slave surface, utilizing a small slip contact definition due to minimal relative sliding or rotation.

**Fig.S3B** illustrates the simulation stages for the hierarchical bionic dry adhesive structure with a rough surface. Initially, the test surface was non-contacting, positioned above either the ordinary or hierarchical structure. As the test surface approached, contact occurred, and the adhesive structure experienced pressure during the contact stage. This continues until maximum pre-pressure was reached, followed by the test surface moving away, transitioning from pressure to tension until maximum tension and therefore maximum adhesive force was achieved, leading to the de-attachment stage. Throughout this process, normal force, strain energy, fracture energy, and maximum contact area were analyzed.


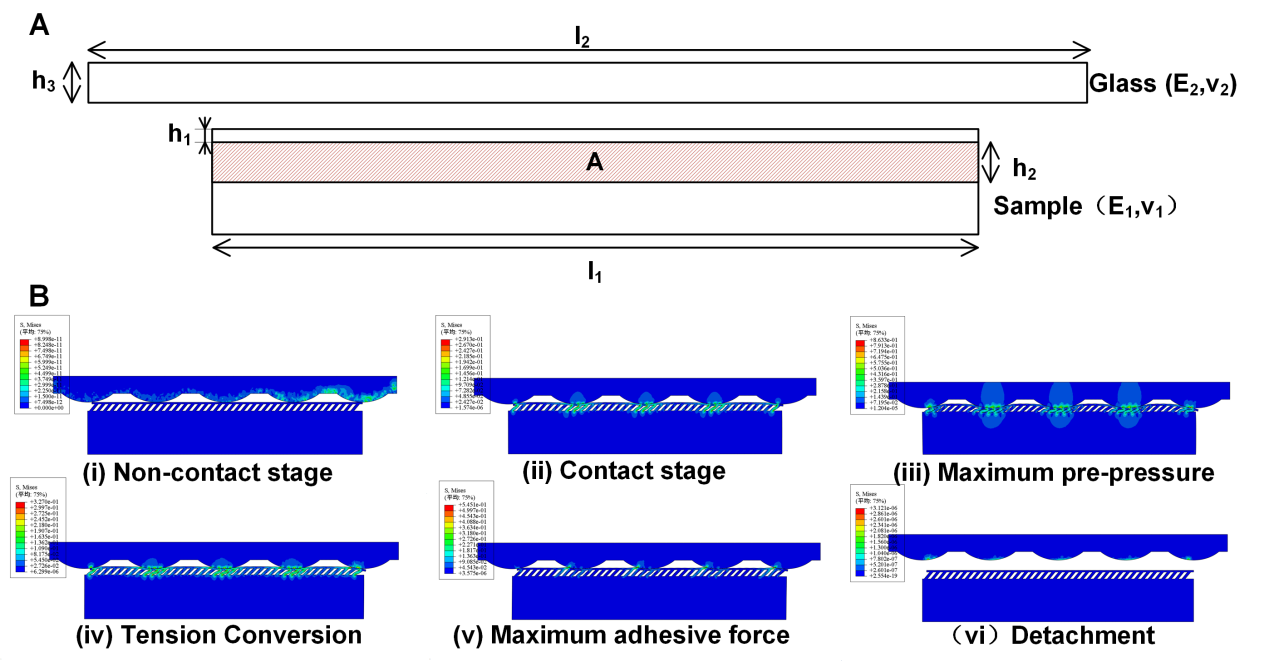


**Fig.S3** Simulation modeling of hierarchical bionic dry adhesive structure. (A) schematic of the structure of a multilevel structural mechanic model, the model was divided into two parts: the sample and the probe. (B) schematic visualization of the adhesion and separation process of a hierarchical bionic dry adhesive structure, with the top structure being the test surface (rough surface) and the bottom structure being either a normal bionic dry adhesive structure or a hierarchical bionic dry adhesive structure (in this case is a hierarchical bionic dry adhesive structure).

Section S4. Characterization of adhesion properties of hierarchical bionic dry adhesive structures on rough surfaces.

In this paper, samples from three different machining methods were tested: planning, flat milling and end milling. Four surface roughness were obtained for each of the machining methods. Since the machined samples were too large to be placed directly on the end of the probe, the NOA light-curing adhesive was used as the testing surface by molding the samples with the same shape but with thickness, volume and mass much smaller than that of the machined samples, which were prepared by the following method. Firstly, custom-made machined standard roughness comparison samples were made, such as **Fig.S4A(I)**. After that, a metal surface counter form mold made of PDMS was prepared by a molding process, as **Fig.S4A(II)**. Finally, the counter-type mold was turned over using NOA to obtain a surface made of NOA with the same properties as the machined surface, as in **Fig.S4A(III)**.

After preparation, the first step was to use a laser confocal microscope to measure the roughness data of each inspection surface. **Fig.S4B-D** are confocal features of planning samples, flat milling samples and end milling samples of Ra = 0.8 μm, Ra = 1.6 μm, Ra = 3.2 μm and Ra = 6.3 μm. In this paper, the accepted contour arithmetic mean deviation Ra and contour maximum height Rz are used to characterize the roughness of the test surface. Measuring by confocal microscopes, **Table S1-S3** show the surface roughness of the standard machined test surfaces of planning, flat milling and end milling, respectively:

**Table S1.** Surface roughness parameters of planning samples.

| Standard Machining Accuracy  Ra (μm) | Arithmetic mean deviation of actual profile  Ra (μm) | Maximum height of actual profile  Rz (μm) |
| --- | --- | --- |
| 0.8 | 0.673 | 4.422 |
| 1.6 | 1.523 | 17.942 |
| 3.2 | 3.335 | 28.883 |
| 6.3 | 6.304 | 38.572 |

**Table S2.** Surface roughness parameters of flat milling samples.

| Standard Machining Accuracy  Ra (μm) | Arithmetic mean deviation of actual profile  Ra (μm) | Maximum height of actual profile  Rz (μm) |
| --- | --- | --- |
| 0.8 | 0.633 | 4.219 |
| 1.6 | 1.443 | 17.332 |
| 3.2 | 3.661 | 29.421 |
| 6.3 | 6.228 | 38.251 |

**Table S3.** Surface roughness parameters of end milling sample.

| Standard Machining Accuracy  Ra (μm) | Arithmetic mean deviation of actual profile  Ra (μm) | Maximum height of actual profile  Rz (μm) |
| --- | --- | --- |
| 0.8 | 0.723 | 4.021 |
| 1.6 | 1.421 | 18.045 |
| 3.2 | 3.753 | 28.561 |
| 6.3 | 6.374 | 39.415 |


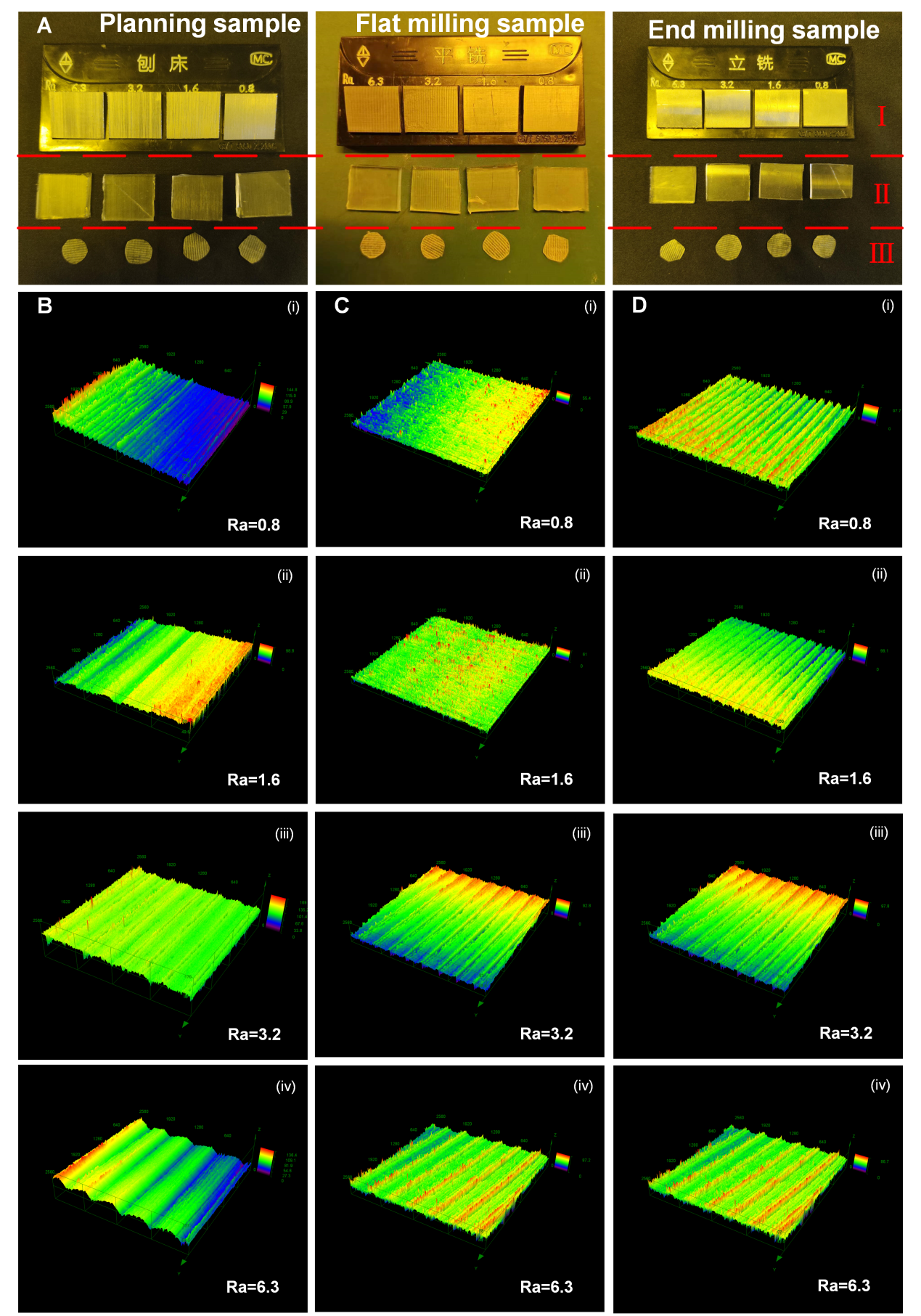


**Fig.S4** Roughness samples and their roughness characterization. (A) standard roughness samples. (I) machined standard roughness comparison sample. (II) reverse mold of the machined sample of PDMS material prepared by molding process. (III) sample of NOA material with machined surface properties obtained by turning the mold again. (B-D) confocal features of planning samples, flat milling samples and end milling samples, including Ra = 0.8 μm, Ra = 1.6 μm, Ra = 3.3 μm and Ra = 6.3 μm.

After preparing the machined surface samples, the LP adhesive force test method was used to test the adhesive force of three different machined roughness surface samples to compare the adhesion effects of the hierarchical bionic dry adhesive structure and the normal bionic dry adhesive structure on daily engineering applications.

**Fig.S5A** shows the adhesion results of standard machined roughness surfaces using the planer method. The inhomogeneous machined surface (Fig.S4A) led to lower precision and reduced adhesion for normal bionic dry adhesive structures. The maximum adhesive force for the normal structure at Ra = 0.8 μm was only 13KPa, indicating a significant loss of adhesion. In contrast, the hierarchical bionic dry adhesive structure achieved 62 KPa under similar conditions. At Ra = 1.6 μm, the hierarchical structure maintained a maximum force of 51 KPa, while the normal structure only reached 22 KPa. At Ra = 3.2μm, the hierarchical structure's maximum force was 61 KPa compared to 40 KPa of the normal one, indicating an improved adhesion effect. However, at Ra = 6.3 μm, the hierarchical structure dropped to 48 KPa, while the normal structure stayed at 26 KPa, showing a decline in effective adhesion as roughness increases. The reason why the adhesive force on planning samples increased and then decreased with roughness was due to the highly uneven surface of the samples with visible tool marks as a result of the characteristics of the planning process. When the surface roughness was relatively low, the tool marks were not only highly undulating in height, but also the spacing between the tool marks was small, which led to a smaller effective contact area for interfacial interaction. As a result, the adhesion performance of the adhesive structures decreased as the surface roughness of the sample decreased. As the surface roughness increased, the spacing between the knife patterns became wider and the high and low undulations of the knife patterns became less pronounced, thus slightly improving the adhesion performance. Until the roughness was so great that the adhesive structure was not able to make good contact with the sample surface, so the adhesion decreased again.

**Fig.S5B** presents the adhesion results for standard machined roughness surfaces from the flat milling method. The hierarchical adhesive structure outperformed the normal bionic dry adhesive structure, achieving a maximum adhesive force of 92 KPa at Ra = 0.8 μm, compared to 80 KPa for the normal structure. As surface roughness increased, both structures experienced a decline in adhesive force; however, this reduction was more significant for the normal structure, which dropped to 30 KPa at Ra = 6.3 μm, rendering it unsuitable for engineering applications. In contrast, the hierarchical structure maintained a force of 65 KPa at Ra = 6.3 μm, down only 29.3% from Ra = 0.8 μm, indicating it remained viable for engineering use.

**Fig.S5C** presents the adhesion results for surface samples machined in end milling mode. At Ra = 0.8 μm, the hierarchical adhesive structure achieved an adhesive force of 83 KPa, slightly higher than the ordinary bionic dry adhesive structure at 80 KPa, making them suitable for most working environments. As surface roughness increased, the ordinary structure’s adhesion initially declined to 55 KPa at Ra = 1.6 μm (31.3% lower than at Ra = 0.8 μm), then rises to 67 KPa at Ra = 3.2 μm and reached to 80 KPa at Ra = 6.3 μm. In contrast, the hierarchical structure showed an increasing trend with adhesive forces of 93 KPa at Ra = 1.6 μm, 90 KPa at Ra = 3.2 μm, and 95 KPa at Ra = 6.3 μm, indicating greater stability.


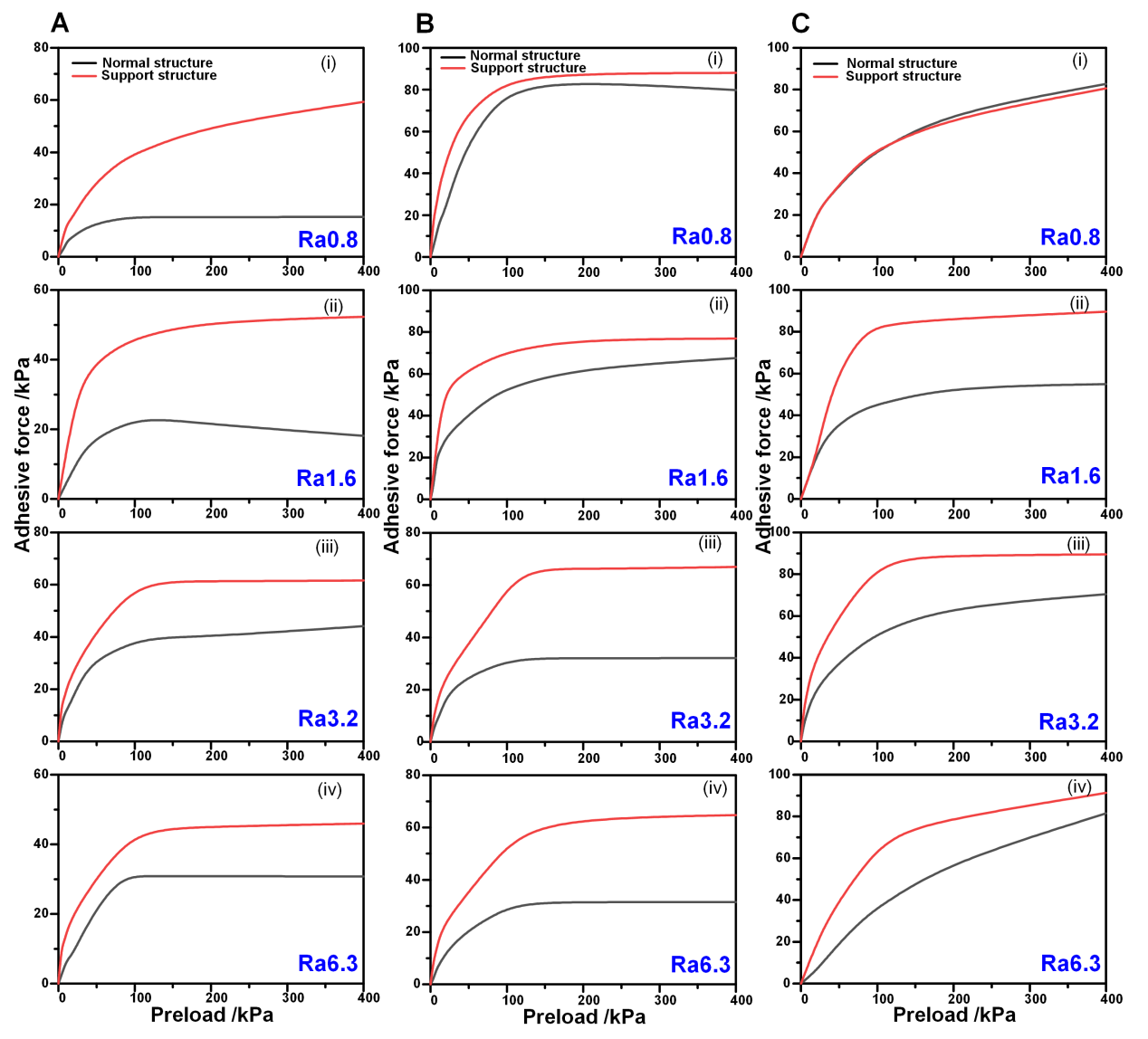


**Fig.S5** Adhesion performance of intelligent adhesive structure on rough surface. (A-C) comparison of the adhesion performance of hierarchical bionic dry adhesive structures and normal bionic dry adhesive structures on planning samples, flat milling samples and end milling samples.

Section S5. The comparison of adhesion performance between normal adhesive structure and hierarchical adhesive structure.

The introduction of inclined supporting micro-pillars enabled the hierarchical bionic dry adhesive structures to exhibit self-regulation of interface contact, thereby enhancing their surface adaptability. Under external forces, hierarchical bionic dry adhesive structures could automatically adjust their shape to optimize contact with the surface, improving adhesive performance. This phenomenon could be observed through optical electron microscopy, as shown in **Fig.S6A**. When hierarchical bionic adhesive structures and normal bionic dry adhesive structures were brought into contact with a surface of transparent glass with irregularities, both structures initially exhibited uniform deformation. However, as pressure increased, the mushroom-shaped bionic dry adhesive structures in the normal adhesive structures lost their optimal contact state, especially under thicker layers of glass. In contrast, the hierarchical bionic dry adhesive structure, under the influence of the inclined supporting structure (which bent under applied force), underwent morphological compensation, allowing the mushroom-shaped micro-pillars to maintain their normal shape and contact state, thereby ensuring the proper functioning of adhesion.

Similarly, when facing a rough surface, the ordinary bionic dry adhesive structure had a lower interfacial adjustment performance, which led to a decrease in the effective contact area, resulting in lower adhesive force. In contrast, the hierarchical bionic dry adhesive structure had better interfacial adjustment performance, and the pillar structure provided morphological compensation, which allowed more individual mushroom-shaped micro-pillars to contribute. This increased the effective contact area, improving the adhesive force. To verify that the hierarchical bionic dry adhesive structure also exhibited better adhesion performance on irregular rough surfaces, the PLA (polylactic acid) sheet (**Fig.S6B**) was chosen as the test sample in this study, with the area in the red box in the figure being the test surface. The arithmetic mean deviation of the actual profile of the PLA plate, obtained through confocal microscopy measurements, was 3.790 μm, and the maximum height of the actual profile was 187.233 μm. **Fig.S6C** shows the comparison of the adhesive force between the hierarchical bionic dry adhesive structure and the normal bionic dry adhesive structure on the surface of the PLA plate. The results indicated that the hierarchical bionic dry adhesive structure still exhibited better adhesion performance when facing irregular and rough surfaces, which was consistent with the theoretical analysis.

After comparing the adhesion performance of the hierarchical bionic dry adhesive structure with that of the normal bionic dry adhesive structure on the regular corrugated rough surface and the uneven rough surface, this paper also compared their performance on smooth planes. Fig.S6D shows the comparison of the adhesive force of the hierarchical bionic dry adhesive structure with that of the normal bionic dry adhesive structure on the smooth glass surface. The results show that the adhesive force of the hierarchical bionic dry adhesive structure on smooth surfaces does not decrease after the introduction of the support layer, but increases slightly. There are two main reasons for this phenomenon: firstly, when subjected to external forces, the support layer of the hierarchical bionic dry adhesive structure has a spring-like effect, which enhances the contact force between the lower layer of the adhesive structure and the target surface through the additional pressure, thus making the contact closer. Secondly, the inclined pillar structure could decompose the vertical force into horizontal components, which can push the mushroom-type adhesive structure to produce horizontal micro-slip in the face of small unevenness of the surface, helping the adhesive structure to "spread" on the surface. This small slip reduces localized contact failures and encourages more areas to conform better to the target surface, thus improving the overall adhesion effect.


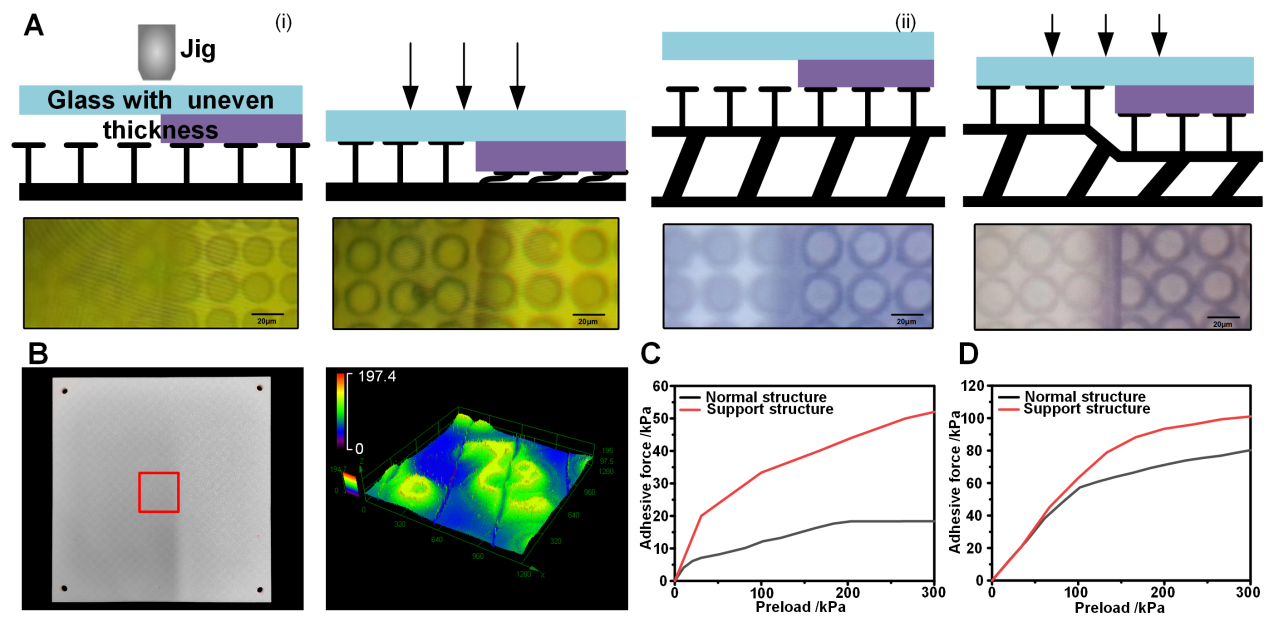


Fig.S6 The comparison of adhesion performance between normal adhesive structure and hierarchical adhesive structure. (A) adhesion effects of normal bionic dry adhesive structures and hierarchical bionic dry adhesive structures at different pressures on transparent uneven glass. (B) the PLA plat and its confocal microscopy measurement image. (C) the adhesive force of the hierarchical bionic adhesive structure and that of the normal bionic adhesive structure on PLA plat. (D) the adhesive force of the hierarchical bionic dry adhesive structure and that of the normal bionic dry adhesive structure on the smooth glass surface

Section S6. The second adhesion-desorption cycle test experiment.

As shown in **Fig.S7**, the results of the second 50 adhesion cycle test of the hierarchical bionic dry adhesive structure are plotted. At the end of the first 50 cycles, the prototype was left for one month to simulate maintenance and upkeep in real-world applications. During this period, the surface of the prototype was cleaned with alcohol and supplemented with a dust-free wipe treatment to remove impurities that may have accumulated and to restore the surface condition. Following this, a second 50 adhesion-desorption cycle test was carried out, which showed that the final maximum adhesion was maintained at 84 kPa, a drop of only 3.4% from the 87 kPa at the start of the cycle; this further emphasizes the positive effect of maintenance measures in prolonging service life. The results of these two tests reveal how the layered bionic dry adhesive structure performs under different conditions of use. What's more, the experiment proves that timely maintenance and care can greatly slow down the decline of adhesion, thus significantly extending the service life of the samples. It is also noteworthy that the maximum adhesion values measured at the end of the two adhesion-desorption cycles were almost identical, which not only verifies the effectiveness of the maintenance measures, but also demonstrates that the structure still has good adhesion properties after multiple uses.


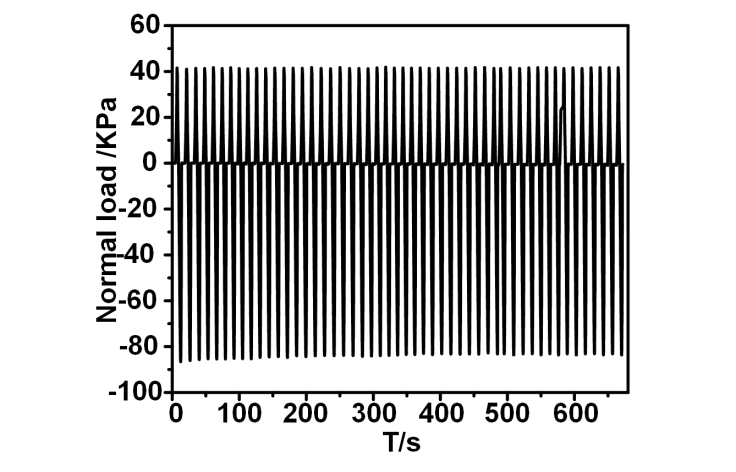


**Fig.S7** The second adhesion-desorption cycle test plot of the adhesive force of the hierarchical bionic dry adhesive structure.

Section S7. Foam backing adhesive structure.

In order to compare the adhesion performance of the hierarchical bionic dry adhesive structure in this paper with that of the foam backing adhesive structure on a rough surface, we fabricated a sponge backing adhesive structure. The mushroom-type bionic adhesive structure of the bottom layer is the same as that used in the hierarchical bionic dry adhesive structure in this paper. The sponge used was 50 ppi (about 0.5 mm pore size) with a thickness of 4 mm. **Fig.S8** shows the SEM image of the sponge-backed adhesive structure.


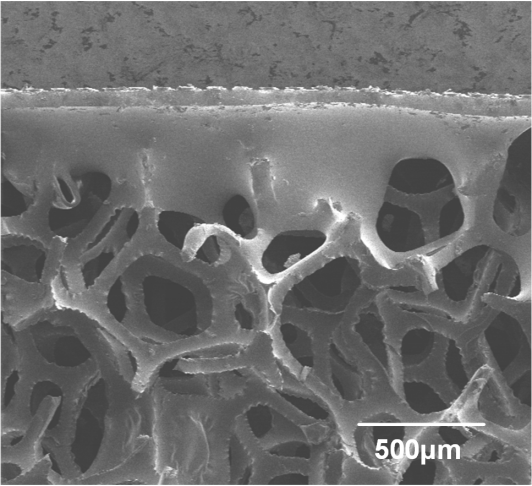


**Fig.S8** The SEM image of the sponge-backed adhesive structure.

Section S8. The comparison of adhesion performance between foam backing adhesive structure and hierarchical adhesive structure.

Apart from PLA plat, PTFE (Polytetrafluoroethylene) plat with regular corrugations was selected for the hierarchical bionic dry adhesive structure and the adhesion sponge backing adhesive structure as the sample for rough surface adhesion testing, and the part in the red box in the figure is the test surface. As **Fig.S9A** shows, the Arithmetic mean deviation of the actual profile of the PLA plat was obtained by confocal microscopy measurements as 4.738 μm. And the maximum height of actual profile was 41.317 μm. **Fig.S9B** is the comparison of adhesion between these two structures on the PTFE plat. when the pre-pressure was 500 kPa, the adhesive force of the hierarchical bionic dry adhesive structure was 31.67 kPa, and that of the sponge backing adhesive structure was 10.50 kPa. Furthermore, **Fig.S9C** is the comparison of adhesion between these two structures on the PLA plat. when the pre-pressure was 500 kPa, the adhesive force of the hierarchical bionic dry adhesive structure was 84.66 kPa, and that of the sponge backing adhesive structure was 71.60 kPa.

This improvement is primarily due to the smaller size of the micro-pillars in the pillar layer of the hierarchical adhesive structure, which enables more effective morphological compensation and results in a significantly larger contact area. In contrast, although the sponge backing, which characterized by its high compressibility and softness, can adapt to surface irregularities to some extent, its inherent flexibility limits its ability to conform closely to surfaces with continuous height variations. This makes it less effective at maintaining consistent contact across complex surfaces. On the other hand, the smaller scale micro-pillars in the hierarchical bionic dry adhesive structure are better suited to accommodate such surface morphology changes. By adjusting their shape in response to surface irregularities, these micro-pillars improve the adhesive structure's ability to adapt, particularly on surfaces with intricate topographies. This design not only increases the overall contact area with the surface but also enhances the adhesive ability to compensate for variations in surface morphology. As a result, the hierarchical bionic dry adhesive structure exhibits superior adhesion strength, particularly on surfaces with complex features or irregularities, offering a significant advantage over the foam backing adhesive systems.


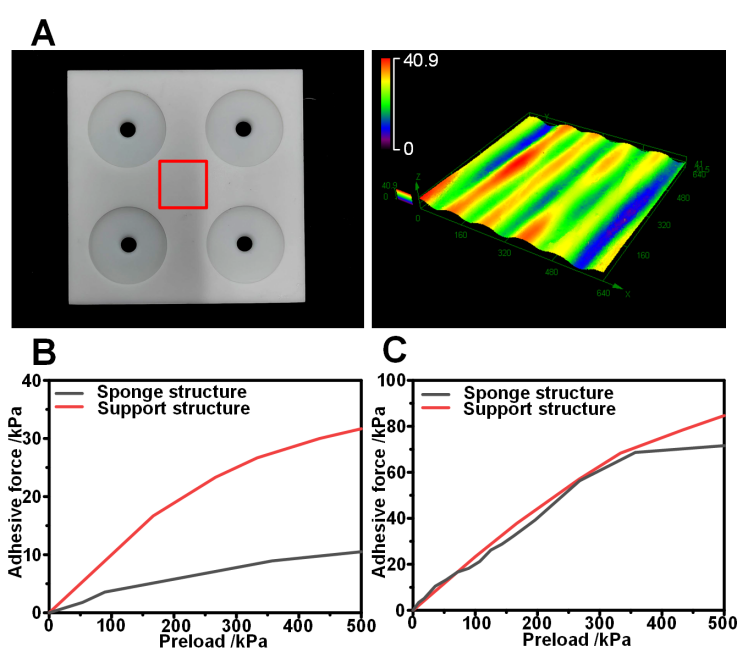


Fig.S9 The comparison of adhesion performance between sponge backing adhesive structure and hierarchical adhesive structure. (A) the PTFE plat and its confocal microscopy measurement image. (B) the adhesive force of the hierarchical bionic adhesive structure and that of the normal bionic adhesive structure on PTFE plat. (D) the adhesive force of the hierarchical bionic dry adhesive structure and that of the normal bionic dry adhesive structure on PLA plat.

Section S9. Testing of regionalized adhesion/sensing integrated structures.

The regionalized adhesion/sensing integrated structure prepared in this paper has 3×3 sensing arrays, and nine signal values could be derived by marking each exported lead separately, labeling each individual array with a name and recording the data. Where the naming of each individual array is shown in **Table S4**. The capacitance value at each point is denoted by *C* and *ΔC* denotes the capacitance change value.

**Table S4.** Array name corresponding location table.

|  | A | B | C |
| --- | --- | --- | --- |
| a | （A，a）= C_11_ | （B，a）= C_21_ | （C，a）= C_31_ |
| b | （A，b）= C_12_ | （B，b）= C_22_ | （C，b）= C_32_ |
| c | （A，c）= C_13_ | （B，c）= C_23_ | （C，c）= C_33_ |

Section S10. Physical testing of interfacial shape sensing of regionalized intelligent bionic dry adhesive structures.

**Fig.S10A** demonstrates the application of the regionalized smart adhesion mechanism and the interface perception function test, which is the behavioral process of the robotic arm in the inverted ink bottle pickup experiment. First, the intelligent adhesive structure was controlled to approach the target surface by controlling the robotic arm, and when the contact between the target surface and the robot was made, the controlling machinery first stopped for a period of time and then carried out a reverse motion to pick up the inverted ink bottle and translated it to the right side, which simulated the pickup and transport processes in the production line. A multiple capacitive signal collector was used to collect the capacitive signal of the regionalized intelligent adhesive structure, and the results are presented in **Fig.S10B**. Since the glass surface at the ink bottle’s back was concave, the signals of the three sensors in the middle did not fluctuate significantly. First, the mechanical arm pressed down to bear the pressure, and then, the mechanical arm paused and still bore the pressure. Next, the mechanical arm rose up slowly, and the pulling force was transformed. The change in the capacitance value could correspond with the simulation of the picking up and transporting processes, when the mechanical arm pressed down and started to touch the bottom surface of the ink bottle. Due to the oval shape of the bottom of the ink bottle; the three sensors in the center were not subjected to normal stresses and therefore the capacitance change value is almost zero, while the other sensors have positive and continuously increasing capacitance change values. Because the two sides of the oval shape were shallower, the two sensors of (b, A) and (b, C) were subjected to less pressure, and the change in the capacitance value was almost zero until the mechanical arm paused the movement, when the capacitance change started to stabilize. Afterward, the ink bottle was lifted, and the capacitance change of the other capacitors, except the middle three, continued to decrease to less than zero. **Fig.S10C** shows the plot of the sensor array during the short stopping period after the control robot arm was pressed down to fully contact the sample. The results indicated that the middle three sensors were not in contact with the target surface, and the three sensors on either side of it, which were close to the middle, were under less pressure; therefore, the capacitance change was small.


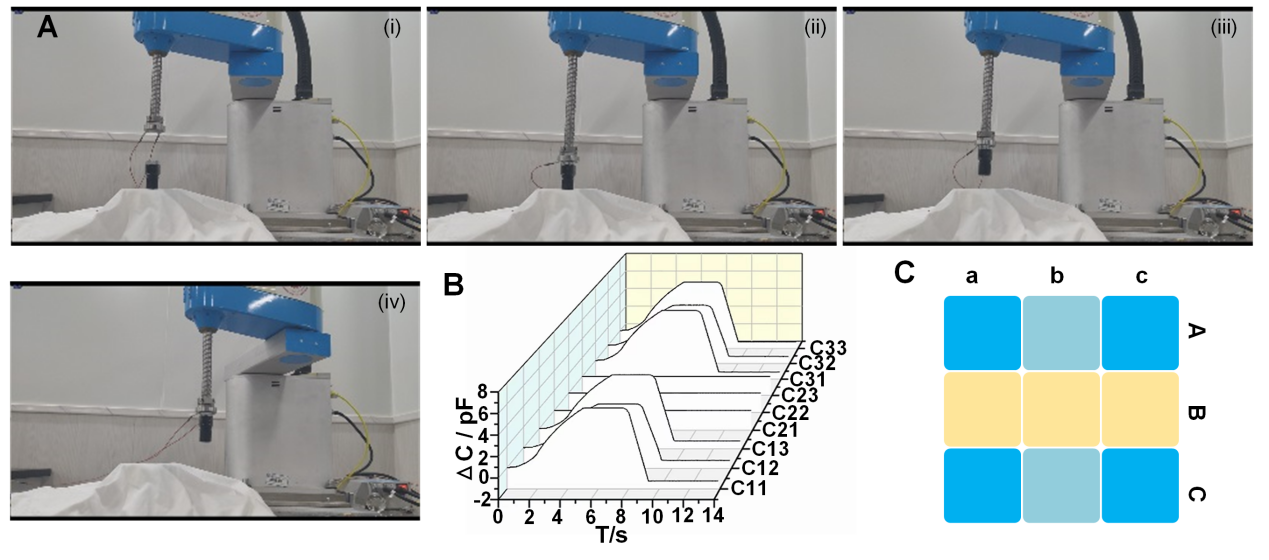


**Fig.S10** Inverted ink bottle pick-up experiment. (A) the inverted ink bottle pickup experimental procedure. (B) the capacitance changes during the inverted ink bottle pickup experiment. (C) the response diagram of the sensing array.

Section S11. Movie.

**Movie S1.** Simulated adhesion process of ordinary bionic dry adhesive structures and rough surfaces.

**Movie S2.** Simulated adhesion process of hierarchical bionic dry adhesive structures and rough surfaces.
